# Homologous recombination-mediated irreversible genome damage underlies telomere-induced senescence

**DOI:** 10.1093/nar/gkab965

**Published:** 2021-11-02

**Authors:** Sabrina Ghadaouia, Marc-Alexandre Olivier, Aurélie Martinez, Tibila Kientega, Jian Qin, Patrick Lambert-Lanteigne, Guillaume B Cardin, Chantal Autexier, Nicolas Malaquin, Francis Rodier

**Affiliations:** Centre de recherche du Centre hospitalier de l’Université de Montréal (CRCHUM), Montreal, QC, H2X 0A9, Canada; Institut du cancer de Montréal, Montreal, QC, H2X 0A9, Canada; Centre de recherche du Centre hospitalier de l’Université de Montréal (CRCHUM), Montreal, QC, H2X 0A9, Canada; Institut du cancer de Montréal, Montreal, QC, H2X 0A9, Canada; Centre de recherche du Centre hospitalier de l’Université de Montréal (CRCHUM), Montreal, QC, H2X 0A9, Canada; Institut du cancer de Montréal, Montreal, QC, H2X 0A9, Canada; Centre de recherche du Centre hospitalier de l’Université de Montréal (CRCHUM), Montreal, QC, H2X 0A9, Canada; Institut du cancer de Montréal, Montreal, QC, H2X 0A9, Canada; Department of Anatomy and Cell Biology, McGill University, Montreal, QC, H3A 0C7, Canada; Jewish General Hospital, Lady Davis Institute, Montreal, QC, H3T 1E2, Canada; Jewish General Hospital, Lady Davis Institute, Montreal, QC, H3T 1E2, Canada; Centre de recherche du Centre hospitalier de l’Université de Montréal (CRCHUM), Montreal, QC, H2X 0A9, Canada; Institut du cancer de Montréal, Montreal, QC, H2X 0A9, Canada; Department of Anatomy and Cell Biology, McGill University, Montreal, QC, H3A 0C7, Canada; Jewish General Hospital, Lady Davis Institute, Montreal, QC, H3T 1E2, Canada; Centre de recherche du Centre hospitalier de l’Université de Montréal (CRCHUM), Montreal, QC, H2X 0A9, Canada; Institut du cancer de Montréal, Montreal, QC, H2X 0A9, Canada; Centre de recherche du Centre hospitalier de l’Université de Montréal (CRCHUM), Montreal, QC, H2X 0A9, Canada; Institut du cancer de Montréal, Montreal, QC, H2X 0A9, Canada; Department of Radiology, Radio-Oncology and Nuclear Medicine, Université de Montréal, Montreal, QC, H3T 1J4, Canada

## Abstract

Loss of telomeric DNA leads to telomere uncapping, which triggers a persistent, p53-centric DNA damage response that sustains a stable senescence-associated proliferation arrest. Here, we show that in normal cells telomere uncapping triggers a focal telomeric DNA damage response accompanied by a transient cell cycle arrest. Subsequent cell division with dysfunctional telomeres resulted in sporadic telomeric sister chromatid fusions that gave rise to next-mitosis genome instability, including non-telomeric DNA lesions responsible for a stable, p53-mediated, senescence-associated proliferation arrest. Unexpectedly, the blocking of Rad51/RPA-mediated homologous recombination, but not non-homologous end joining (NHEJ), prevented senescence despite multiple dysfunctional telomeres. When cells approached natural replicative senescence, interphase senescent cells displayed genome instability, whereas near-senescent cells that underwent mitosis despite the presence of uncapped telomeres did not. This suggests that these near-senescent cells had not yet acquired irreversible telomeric fusions. We propose a new model for telomere-initiated senescence where tolerance of telomere uncapping eventually results in irreversible non-telomeric DNA lesions leading to stable senescence. Paradoxically, our work reveals that senescence-associated tumor suppression from telomere shortening requires irreversible genome instability at the single-cell level, which suggests that interventions to repair telomeres in the pre-senescent state could prevent senescence and genome instability.

## Graphical Abstract

**Graphical Abstract:**
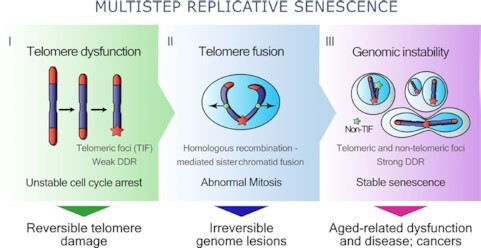
Replicative senescence is orchestrated by a multi-step mechanism, exploiting homologous-directed DNA-repair at short telomeres to fuse sister chromatids generating irreparable genome damage and stable proliferative arrest. Paradoxically, senescence requires genome instability to ensure irreversibility.

## INTRODUCTION

Telomeres are non-coding repetitive DNA sequences at the extremity of chromosomes that protect free DNA ends from exonuclease-mediated degradation or end-to-end fusions (1). In the absence of telomerase, telomeres shorten at each cell division until they become dysfunctional, activating a DNA damage response (DDR) that arrests cell proliferation and induces replicative senescence (2–5). This stable senescence-associated proliferation arrest prevents cells with unstable genomes from multiplying and acts as a key mechanism to suppress cancer (6). While senescent cells have broad physiological roles that initiate and orchestrate tissue repair responses (7–12), the accumulation of these cells and those driven by telomere dysfunction can degrade tissue functions, thereby linking cellular senescence to aging and cancer (12–15).

Telomeric protection is provided by the shelterin complex composed of six protein subunits (TRF1, TRF2, Tin2, Rap1, POT1 and TPP1). These assemble on telomeric DNA repeats and force the telomeres into a protective t-loop structure to hide the single-stranded telomeric DNA overhang (16,17). When shortened telomeres cannot form a proper t-loop, telomere uncapping (TU) occurs, and the dysfunctional telomere is recognized as a DNA double-strand break (DSB). This initiates the formation of telomere-induced DNA damage foci (TIF), first described as telomere dysfunction-induced focus (4), that contain the DSB-associated factors 53BP1, phosphorylated histone variant H2AX (γH2AX) or activated ATM, which together serve to propagate DDR signaling (2,16). Because uncapped telomeres are irreparable, DDR signaling persists and sustains p53-enforced cell cycle checkpoints, which will inhibit cell proliferation and establish senescence (2–4,18–20). This telomere-mediated senescence mechanism is proposed to be a powerful suppressor of genomic instability that prevents cancer (21). Although inactivation of key DDR components such as ATM, CHK2 or p53 can delay or transiently reverse senescence (2,3,22,23), telomerase overexpression for telomere repair cannot reverse established senescence since telomere extension requires active S-phase DNA synthesis (23,24).

A critical gap in understanding telomere-initiated senescence is the exact cellular threshold for a number of dysfunctional telomeres to establish proliferative arrest via a persistent DDR signal. Also unknown is why p53 is not stably activated by a single TU event, which is considered an irreparable DSB (18–20). In yeast, a single short telomere can initiate senescence but can also lead to DNA damage adaptation and genome instability (25–27). In contrast, at least 5–10 dysfunctional telomeres appear necessary to trigger replicative senescence in normal mammalian cells (28–31). The average human telomere length prior to senescence is 1–2 kb, but mammalian telomeres shorter than 1 kb have been observed in proliferating cells (28,29,32), suggesting that mammalian cells can tolerate a certain number of dysfunctional telomeres. Apart from a DDR threshold, another trigger for senescence may be revealed from the accumulation of critically short dysfunctional telomeres in proliferating p53-negative cells. The telomere crisis under this setting can potentiate non-homologous end joining (NHEJ)-mediated end-to-end telomeric fusions between different chromosomes, or alt-NHEJ-mediated sister chromatid fusions, which initiate breakage–fusion–bridge cycles that give rise to genome instability (1,33–39). Almost all precedent work on telomere-mediated instability has focused on the bypass of senescence following TU in the absence of p53. It is still unclear why a DDR threshold would regulate senescence in normal cells that harbor dysfunctional telomeres, while a telomere crisis would happen in dividing p53-deficient cells. One current hypothesis is that uncapping in normal cells would open telomeres in their intermediate state and allow TIF formation, but would still prevent NHEJ-mediated end-to-end telomeric fusions and polyploidy, thus preserving genome stability (40). Alternatively, sustained proliferation in p53-deficient cells would further shorten telomeres, allow their complete opening and result in the fusions leading to breakage–fusion–bridge cycles and genomic instability (41,42). However, the idea that telomere-mediated senescence yields p53-positive normal cells with a stable genome is inconsistent with several reports of replicative senescence associating with genomic instability (43–50). And at least one observation suggests that some TRF2 mutants can disrupt shelterin in normal fibroblasts with wild-type p53 to generate genome instability (51,52).

In this study, we examined whether normal human cells could tolerate TU and showed that p53 wild-type diploid cells reacted but rapidly adapted to TU. We performed controlled inactivation of essential shelterin complex proteins to induce TU and telomeric DDR in normal cells (1,53,54). Although t-loop disruption is the accepted trigger for a telomeric DDR that leads to senescence (51), we demonstrated that entry into senescence rather involved DNA repair mechanisms and a multistep relationship between telomeric and non-telomeric irreversible damage, which together control a transient unstable (pre-senescence) state and a stable (senescence) proliferative arrest. In contrast to previous senescence models, we suggest that irreversible genomic instability, rather than TIF, is required to establish replicative senescence in normal cells.

## MATERIALS AND METHODS

### Cell culture and treatments

Primary HCA2 and BJ human foreskin fibroblasts were given by J. Smith (University of Texas, San Antonio) and cultured under ∼21% oxygen (ambient levels) in Dulbecco’s modified Eagle medium supplemented with 8% fetal bovine serum (FBS), 2.5 μg/ml fungizone and 100 U/ml penicillin/streptomycin. Under serum starvation (0.1% FBS) conditions, medium was changed 24 h prior to experiments and dishes were rinsed three times with low-serum medium. For all experiments, medium was changed every 2 days (except for viability assays with DRAQ7 to keep floating dead cells). Unless specified, early passage fibroblasts [defined by a population doubling (PD) <35 and a 24-h 5-ethynyl-2′-deoxyuridine (EdU) labeling index of >75%] were used in all experiments. PD of primary cells was determined with the following equation: current PD = last PD + 3.32 log_2_(cell number/cells seeded). Cell populations were considered to be in replicative senescence when they presented a 24-h EdU labeling index of <5% and were positive for senescence-associated β-galactosidase (SA-B-GAL) activity (approximately PD70 for BJ and HCA2 cells). All lentiviruses used for infections were generated in 293FT packaging cells (Invitrogen) as previously described (55). Where indicated, cells were treated for 4 h with 100 ng/ml of nocodazole (Sigma) or with 30 ng/ml of sustained doxycycline (Dox; Sigma). For experiments with Ku-55933 [ATM inhibitor (ATMi); Sigma], cells were treated with 5, 10 or 20 μM of the inhibitor 24 h before and sustained throughout the experiment. Dox and ATMi treatments were changed every 2 days, and when Dox was removed during experiments, dishes were rinsed three times with fresh medium. Where mentioned, 1 μM of EdU was added 24 h prior to daily fixation (24-h EdU pulses), as well as 10 μM of bromodeoxyuridine (BrdU) 24 or 72 h prior to fixation (24- or 72-h BrdU pulses).

### Viruses and infections

Viruses were generated as described previously (55,56). Titers were adjusted to achieve ∼90% infectivity with 4 μg/ml of polybrene in a final volume of 1 ml in six-well plates (Falcon). Lentiviruses encoding short hairpin RNAs (shRNAs) in pLKO.1–TRC cloning vectors against GFP, Ku80, Ligase IV, RPA and Rad51 were purchased from Open Biosystems. shRNA sequences against Tin2, TRF2 and POT1 were synthetized by GeneArt (Invitrogen) into pENTR/H1/TO and were inserted in the w17-1 destination vector from Dr Eric Campeau lentivector library (56); all entry–destination vectors used are from this reference and available at Addgene. shRNA sequences against GFP and p53 were cloned, respectively, into 728-2 and 430-1 entry vectors and inserted in the w17-1 destination vector. All shRNA target sequences are provided in Supplementary Table S1. Tin2DN [pIRES2-EGFP bicistronic vector; previously referred to as Tin2-15C (57,58)] was inserted into 670-1 (pLenti CMV/TO puro) destination vector. Tet repressor (TetR) in pENTR1A (559-1) was a gift from Eric Campeau (Addgene plasmid #22265) and was inserted in a 706-1 (pLenti CMV blast) destination vector. The inducible p16 lentivector was described previously (56,59). Finally, H2B-GFP from pENTR1A-H2B-GFP (60) was inserted into the W530-1 (pLenti PGK Hygro) destination vector. Cells were selected 48 h following infection using 1 μg/ml of puromycin, 1 μg/ml of blasticidin or 100 μg/ml of hygromycin.

### Irradiation

Where indicated, cells were X-irradiated at rates of ≥0.75 Gy/min using Gammacell^®^ 3000 irradiator Elan, until reaching a final dose of 1 or 4 Gy (total).

### Live imaging-based cell proliferation and mitosis duration analysis

Cells were infected or not with lentiviruses expressing H2B-GFP, seeded in six-well plates (Falcon), treated as described and incubated in the IncuCyte™ S3 Live-Cell Analysis System (IncuCyte HD, Essen Bioscience). Pictures were taken at 2-h intervals in three separate regions/well using a 10× objective in phase contrast and green fluorescence. Cell proliferation was assessed for each condition using IncuCyte™ S3 software from confluence mask or H2B-GFP cell nuclei number measurement. For mitosis, pictures were taken at 15-min intervals in three separate regions/well using a 20× objective in green fluorescence. Durations of mitosis were determined from the moment of chromatin condensation until the formation of two nuclei.

### SA-B-GAL detection

SA-B-GAL assay was performed as previously described (61). Briefly, cells were cultured in six-well plates (Falcon), washed once with 1× phosphate-buffered saline (PBS) and fixed with 5% formalin (Sigma) for 4 min. Cells were washed with 1× PBS and incubated at 37°C for 16 h in the SA-B-GAL staining solution containing 1 mg/ml of 5-bromo-4-chloro-3-indolyl-β-d-galactopyranoside (Invitrogen) in dimethyl sulfoxide (DMSO, 20 mg/ml stock), 5 mM of potassium ferricyanide, 150 mM of NaCl, 2 mM of MgCl_2_ and 40 mM of citric acid/sodium phosphate at pH 6.0. The next day, cells were washed once with methanol, twice with 1× PBS and nuclei were stained using DAPI (0.5 μg/ml) in 1× PBS. Cells were washed again twice with 1× PBS. Bright-field and DAPI pictures determined the percentage of positive cells.

### Immunofluorescence

Cells were cultured in black 96-well plates (Corning) or Falcon™ chambered cell culture slides (four- or eight-well, Nunc, Penfield), fixed in 10% formalin (Sigma) for 10 min at room temperature (RT), washed with 1× PBS and permeabilized in 1× PBS–0.25% Triton™ X-100 (Sigma) for 10 min. Wells or slides were washed with 1× PBS and unspecific antibody binding was blocked for 1 h using 1% bovine serum albumin (BSA, IgG/protease-free; Jackson ImmunoResearch) and 4% normal donkey serum (Sigma) diluted in 1× PBS (blocking solution). Primary antibodies (Supplementary Table S2) were diluted in blocking solution and incubated with fixed cells overnight at 4°C. Cells were washed three times in 1× PBS–0.05% Tween™ 20 (Fisher Scientific) and incubated with secondary antibodies for 1 h at RT. Cells were washed three times in 1× PBS–0.05% Tween™ 20 and slides were mounted with ProLong™ Gold Antifade Reagent with DAPI (Life Technologies), while nuclei of cells in 96-well plates were stained using DAPI solution (0.5 μg/ml) in 1× PBS. Images were acquired with a Carl Zeiss AxioObserver Z1 fluorescence microscope (Jena, Germany) using a 20× objective and quantifications were performed with AxioVision and ImageJ softwares. The number of foci per nucleus was analyzed in >200 nuclei for each condition.

### Fluorescence *in situ* hybridization for telomeric PNA probes in combination with immunofluorescence

Cells were seeded onto coverslips and fixed in 10% formalin (Sigma) for 10 min at RT and permeabilized in 1× PBS–0.25% Triton™ X-100 (Sigma) for 10 min. *Immunofluorescence*: Coverslips were blocked for 1 h in 1× PBG [1× PBS containing 5% BSA IgG/protease-free (Jackson ImmunoResearch) and 2% fish gelatin (Sigma)]. Primary antibodies (Supplementary Table S2) were diluted in 1× PBG and incubated with fixed cells overnight at 4°C. Cells were washed with 1× PBG and incubated with secondary antibodies for 45 min at RT and then washed again. *Telomeric fluorescencein situ hybridization* (*tFISH*): Cells were re-fixed with 10% formalin–0.1% Triton for 10 min and incubated with 10 mM glycine for 30 min, all at RT. Coverslips were washed and incubated at 80°C for 7 min with a hybridization mixture containing C-rich/leading strand telomere PNA probes (TelC-Cy3, Panagene) diluted in formamide, Tris–HCl (pH 7.4) and 1× blocking reagent (Roche). Hybridization continued for 3 h in sealed humidified boxes at RT. Coverslips were washed several times with 70% formamide and 0.1% BSA in 1% Tris–HCl (pH 7.4) and thereafter with a wash buffer of 0.15 M NaCl and 0.07% Tween 20 (Fisher Scientific) in 10% Tris–HCl (pH 7.4). Coverslips were mounted with ProLong Gold Antifade Reagent with DAPI (Life Technologies) on glass slides. Images were acquired with a Carl Zeiss AxioObserver Z1 fluorescence microscope (Jena, Germany) using a 63× objective with Z-stack systems (nine stacks of 0.240 μm) and quantification was performed with AxioVision and ImageJ softwares. The percentage of metaphase containing sister chromatid, chromatid-type dicentric or chromosome-type dicentric fusions was determined analyzing >30 metaphases for each condition. The number of foci per nucleus was analyzed in >100 nuclei for each condition. The mean fluorescence intensity of telomeric PNA probes was measured analyzing >30 nuclei for each condition.

### Telomere restriction fragment analysis

Analysis of telomere length was performed as previously described (62), with a few modifications. Briefly, genomic DNA extracted from HCA2 primary foreskin fibroblasts was digested overnight with HinfI/RsaI (NEB). Two micrograms of the digested DNA was electrophoresed in a 1% agarose gel in 0.5× TBE, on a pulse field electrophoresis gel apparatus (CHEF-DR II System, Bio-Rad) with the following parameters: 5 V/cm, 10 h, initial switch time 1 s, final switch time 6 s. The gel was quickly washed in water, denatured for 30 min in 0.5 N NaOH and 1.5 M NaCl and neutralized for 30 min in 0.5 M Tris–HCl (pH 7.5) and 1.5 M NaCl. The gel was dried at RT for 30 min, and then at 50°C for 1 h. Hybridization was carried out at 37°C overnight, in 5× SSC (75 mM sodium citrate, pH 7.0, 0.75 M NaCl), 5× Denhardt’s solution [0.1% (w/v) BSA, 0.1% (w/v) Ficoll 400, 0.1% (w/v) polyvinylpyrrolidone] and 0.1× P-wash (10 mM Na_2_HPO_4_, 1 mM Na_2_H_2_P_2_O_7_), in the presence of γ-^32^P ATP-labeled (C_3_TA_2_)_3_ probe. Gel was rinsed 2× at RT in 2× SSC, and then washed three times for 10 min with 2× SSC. The gel was exposed on a phosphorimager cassette for 7 days and scanned using the Storm 840 (GE Healthcare). A γ-^32^P ATP-labeled λ DNA-BstEII digest (NEB #N3014) molecular weight marker was used to determine telomere length. Using ImageQuant TL software (GE Healthcare), 30 boxes were created for each sample lane. Telomere length distribution was represented from the telomere restriction fragment (TRF) gel by a plot of telomere length versus percentage of telomeres (intensity).

### FISH for genomic DNA probes

Cells were seeded onto coverslips in 12-well plates (Falcon) and fixed in 10% formalin (Sigma) for 10 min at RT and permeabilized in 1× PBS–0.25% Triton™ X-100 (Sigma) for 10 min. Coverslips were incubated with 100 μg/ml RNase A (Epicentre) at 37°C for 1 h and then with 2× SSC at 75°C for 15 min. Cells were digested in pepsin solution (4 mg/ml in 0.9% NaCl, pH 1.5) for 15 min at 37°C, rinsed in 2× SSC at RT for 5 min and air dried on a 37°C heater. Coverslips were incubated with two labeled fluorescent DNA probes (CEP8-SpectrumAqua and MYC-SpectrumGreen, Vysis, Downers Grove) diluted in hybridization buffer (Vysis, Downers Grove) at 75°C for 3 min. Hybridization continued overnight in sealed humidified boxes at 42°C. Coverslips were washed several times with 0.1% SSC–20% formamide at 37°C for 5 min, 0.1× SSC at 37°C for 15 min and 2× SSC at 37°C for 15 min. Coverslips were mounted with ProLong Gold Antifade Reagent with DAPI (Life Technologies) on glass slides. Images were acquired with a Carl Zeiss AxioObserver Z1 fluorescence microscope (Jena, Germany) using a 63× objective with Z-stack systems (nine stacks of 0.240 μm) and quantification was performed with AxioVision and ImageJ softwares. Percentage of cells containing copy number aberrations was determined analyzing >100 cells for each condition.

### Metaphase spread

Cells were seeded in 100-mm Petri dishes (Falcon) and treated 24 h after as indicated. When cells reached 75% confluence, 1 μM of nocodazole (Sigma) was added to the medium for 4 h. Cells were then trypsinized, centrifuged at 1500 × *g* for 5 min and resuspended in 0.075 M KCl warmed at 37°C, which was carefully added dropwise with a Pasteur pipette to cells under constant agitation. After an incubation of 20 min at 37°C, cells were centrifuged as above, medium was removed and freshly prepared fixative (3:1 methanol:glacial acetic acid) on ice was added dropwise. Centrifugation and fixative step were repeated four times and DNA suspensions were dropped from a height of 10 cm on briefly rinsed glass slides (in fixative). Fixative was added to bath the cells, and the slides were allowed to air dry. tFISH staining was performed when indicated.

### Flow cytometry for cell cycle and cell death analysis

Cells were cultured in six-well plates (Falcon), trypsinized, centrifuged at 1500 × *g* for 5 min and then washed three times in 1× PBS. *Cell cycle analysis*: Live cells were fixed and permeabilized for 24 h using 70% ethanol. Cells were then incubated for 30 min at RT with 100 μg/ml RNase A and 25 μg/ml propidium iodide. *Cell death analysis*: Cells kept throughout the duration of the experiments were incubated and stained with 0.9 nM of DRAQ7 (ab109202, Abcam) during 30 min at RT. *Analysis*: Cells were sorted according to their propidium iodide and DRAQ7 intensity with fluorescence-activated cell sorting (FACS). For each condition, a maximum of 10 000 events were counted. FACS was performed using a Fortessa flow cytometer (BD Biosciences, Mississauga, ON) and the analysis performed with FlowJo software.

### EdU detection

Cells were cultured in black 96-well plates (Corning) or 4-well Falcon™ chambered cell culture slides (Nunc, Penfield), and pulsed with 1 μM of EdU for 24 h, and then fixed in 10% formalin (Sigma). EdU was labeled using Click-iT protocol (Invitrogen) with Alexa Fluor 647 dye. Slides were washed and mounted with ProLong Gold Antifade Reagent with DAPI (Life Technologies). Images were acquired with a Carl Zeiss AxioObserver Z1 fluorescence microscope (Jena, Germany) using a 20× objective and quantification was performed with AxioVision and ImageJ softwares. Percentage of EdU-positive cells was measured in >200 nuclei for each condition.

### BrdU detection

Cells were cultured in four-well Falcon™ chambered cell culture slides (Nunc, Penfield) and pulsed with 10 μM of BrdU for 24 or 72 h, and then fixed in 10% formalin (Sigma) for 10 min at RT and permeabilized in 1× PBS–0.25% Triton™ X-100 (Sigma) for 10 min. Genomic double-stranded DNA was then enzymatically denatured using 10 U/ml DNase I (Roche) and 400 U/ml EXO III (New England Biolabs) for 30 min at 37°C. To stain BrdU, the previously described immunofluorescence protocol was used.

### Clonogenic assay

After indicated treatments, single-cell suspensions were trypsinized and cells were counted. Appropriate cell numbers were seeded according to doubling time (∼32 h for BJ cells, 1000 cells per well) in six-well plates (Falcon). To allow colony proliferation, cells were incubated at 37°C for 5 days, and then fixed with 10% formalin (Sigma) for 10 min. Colonies were stained with 0.01% (m/v) crystal violet in ultrapure water for 1 h. Excess crystal violet was washed away with water and dishes were air dried. Stereomicroscope was used to count individual colonies, which were reported as a percentage of the control.

### Neutral comet assay

Cells were seeded in 100-mm Petri dishes (Falcon) and treated 24 h after as indicated. Single-cell suspensions were trypsinized and cells were counted. Ten thousand cells were mixed with LMAgarose (Invitrogen, cat. #4250-050-02) at 37°C and added onto a well on CometSlides™ (Invitrogen, cat. #4250-004-03). Slides were placed at 4°C in the dark for 10 min and immersed in 4°C lysis solution (Invitrogen, cat. #4250-010-01) for 30 min, and then in 4°C 1× neutral electrophoresis buffer (50 mM Tris base and 150 mM sodium acetate trihydrate) for 15 min. Slides were placed in an electrophoresis slide tray and 1 V/cm was applied for 20 min. Slides were immediately immersed in precipitation solution (1 M ammonium acetate in 95% ethanol) and in 70% ethanol, both for 30 min. The samples were dried at 37°C for 15 min and slides were mounted with ProLong Gold Antifade Reagent with DAPI (Life Technologies). Images were acquired with a Carl Zeiss AxioObserver Z1 fluorescence microscope (Jena, Germany) using a 20× objective and quantification was performed with AxioVision and CaspLab softwares for >100 comets per condition.

### Protein preparation and western blot analysis

Cells were seeded in 100-mm Petri dishes (Falcon) and treated 24 h after as indicated. In mammalian protein extraction reagent (Pierce) complete with protease and phosphatase inhibitor cocktail (Sigma), cells were scraped and centrifugated to remove cellular debris. Protein concentrations of whole cell lysates were determined using the bicinchoninic acid protein assay (Pierce); samples were diluted in 5 mM dithiothreitol and Laemmli buffer (final 1×) and heated at 95°C for 5 min. At least 10 μg of total proteins were separated on 7.5% or 4–15% gradient Tris–glycine SDS-polyacrylamide gels (Bio-Rad), and then transferred onto PVDF membranes (Hybond-C Extra, GE Healthcare Life Sciences). Membranes were blocked in 2% BSA (Sigma) in 1× PBS for 1 h at RT. Primary antibodies (Supplementary Table S2) diluted in 2% BSA were incubated with membranes overnight at 4°C. Membranes were washed three times with 1× PBS–0.1% Tween™ 20 (Sigma) and bound antibodies were detected using peroxidase-conjugated secondary antibodies (Santa Cruz), followed by enhanced chemiluminescence (Pierce). Signal was detected using ChemiDoc™ MP Imaging System (Bio-Rad). GAPDH was used to normalize results using the ImageLab 6 Software (Bio-Rad).

### Real-time qPCR

Cells were seeded in 100-mm Petri dishes (Falcon) and treated 24 h after as indicated. Total RNA was isolated with the RNeasy kit (Qiagen) and stored at −80°C. Reverse transcription of 1 μg total RNA was assessed using the Superscript III first-strand reverse transcriptase kit (Invitrogen; 200 units) according to manufacturer’s instructions. Reverse-transcribed solution was diluted 10 times and qPCR was performed using 400 nM of the specific primers and SYBR Green Select Master Mix (Applied Biosystems). Primers for PCR (Supplementary Table S3) were designed with NCBI Primer-BLAST software (http://blast.ncbi.nlm.nih.gov/). The qPCR protocol followed recommendations for the StepOnePlus™ real-time system (Applied Biosystems). PCR amplification products were revealed using KAPA SYBR^®^ fast green fluorescence (ABl Prism™ Kit, Kapa Biosystems). Data analysis was performed using the StepOne™ software (Applied Biosystems). All target gene transcripts were normalized to the expression of TATA-binding protein.

## RESULTS

### Telomere dysfunction induces a transient cell cycle arrest

To probe the cellular responses to TU, we used normal human diploid HCA2 fibroblasts that undergo telomere-mediated, p53-dependent replicative senescence (30). We introduced a Dox-inducible mutant of Tin2 that cannot bind TRF1 (Tin2DN), coupled to an eGFP reporter to confirm Tin2DN expression at the single-cell level (30,54,56–58) (Supplementary Figure S1A–C). Tin2DN reduces TRF1 levels at telomeres and almost completely displaces TRF2 to induce TU (57). As expected, sustained Dox induction of Tin2DN caused shelterin disruption and formation of nuclear 53BP1/γH2AX double-positive DNA damage foci (DDF), which are associated with senescence, as assessed by SA-B-GAL. Alternatively, Dox induction of the p16^INK4a^ cyclin-dependent kinase inhibitor triggered a DDF-free senescence (59) (Supplementary Figure S1D and E).

**Figure 1.**
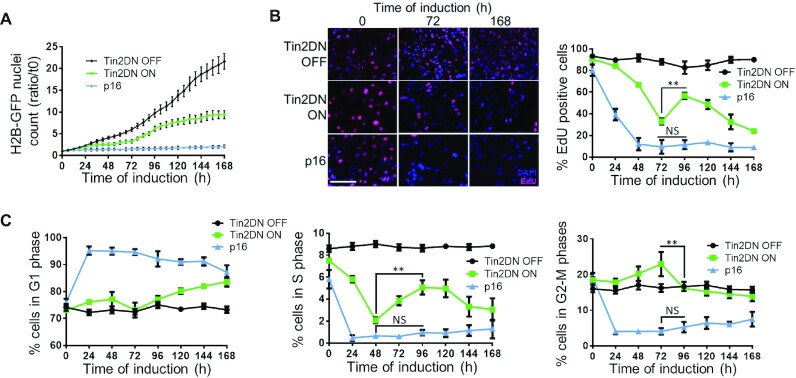
Telomere-induced proliferation arrest is unstable. HCA2 expressing inducible Tin2DN or p16 were treated with Dox (ON) or not (OFF). (**A**) Cells co-infected with H2B-GFP lentivirus were cultured for live cell imaging (IncuCyte). Counts of H2B-GFP-stained nuclei were taken every 6 h and reported against time 0. Scale bar: 200 μm. (**B**) Cells were pulsed with 1 μM of EdU for 24 h (24-h EdU pulses) and stained using immunofluorescence for EdU (red) and DAPI (blue, nuclear counterstain). Graph shows percentage of EdU-positive cells in panel. (**C**) Cells stained with propidium iodide were quantified by flow cytometry. Time courses show percentage of cells in G1 (left panel), S (middle panel) and G2–M (right panel) phases. All quantified data are the mean ± SD of triplicates and represent at least three independent experiments. NS: non-significant. Two-way ANOVA: **P< 0.01.

**Figure 2.**
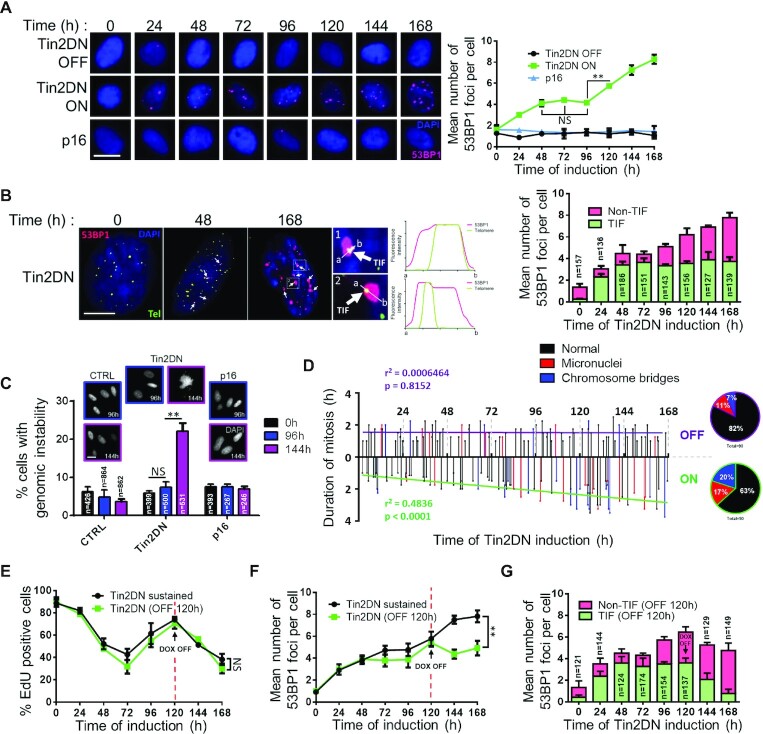
Sustained telomere dysfunction causes non-telomeric DNA breaks and genomic instability. HCA2 expressing inducible Tin2DN, p16 or TetR alone (CTRL) were treated with Dox (ON) or not (OFF) and stained for 53BP1 (red) and DAPI (blue). (**A**) Representative HCA2 cells at each time point (panel) and mean number of 53BP1 foci per nuclei (graph). Scale bar: 20 μm. (**B**) Representative BJ cells during Tin2DN induction (left panels). Telomeres probed using tFISH (green). Foci that colocalized with telomeric probes were TIF (white arrows) and those that did not were non-TIF. Fluorescence intensity for 53BP1 and the telomere was linearly assessed between a and b. Right graph shows mean number of 53BP1 foci per nuclei split into TIF (green) and non-TIF (pink). Scale bar: 10 μm. (**C**) Percentage of DAPI-stained HCA2 cells showing genomic instability (mitotic catastrophes, chromosome bridges or micronuclei) with images of representative HCA2 cells. Scale bar: 20 μm. (**D**) BJ cells infected with H2B-GFP lentivirus were cultured for live cell imaging (IncuCyte) with (ON) or without (OFF) Tin2DN induction. Duration of each mitosis over time was observed at single-cell level for a linear correlation. Circular diagrams indicate percentage of abnormal mitosis. (**E**, **F**) Tin2DN was induced for only 120 h (OFF 120 h, green line) or sustained throughout the experiment (black line). (**E**) Percentage of EdU-positive cells from 24-h EdU pulses. (**F**) Mean number of 53BP1 foci per nuclei. (**G**) Graph shows mean number of 53BP1 foci per nuclei for an ‘OFF 120 h’ experiment split into TIF (green) and non-TIF (pink). All quantified data are the mean ± SD of triplicates and represent at least two independent experiments. NS: non-significant. Two-way ANOVA: **P< 0.01.

To probe the dynamics of entry into senescence following Dox induction, we tagged cells with histone H2B-GFP and performed proliferation assays with live cell imaging. The p16^INK4a^ induction resulted in a rapid and stable proliferation arrest. In contrast, Tin2DN induction resulted in a transient arrest (∼24–72 h post-induction) followed by a short proliferation period before another arrest that coincided with the senescence state (Figure 1A and Supplementary Figure S1F). To validate different cell cycle dynamics between Tin2DN- and p16^INK4a^-induced senescence, we used EdU pulse labeling and FACS analysis (Figure 1B and C). p16^INK4a^-induced cells showed a stable DNA synthesis arrest and accumulated in G1 within 24 h. Alternatively, Tin2DN-induced cells displayed three distinct stages revealing a multistep senescence entry process. In the first 72 h (Tin2DN stage 1), cells reduced their proliferation, consistent with TU induction and DDR–p53 activation, and led to a primary arrest with a decrease in S-phase cells and a slight increase in G1 cells. Tin2DN stage 2 (72–120 h) showed an increase in cells undergoing S phase despite nearly all cells (>98%) harboring >1 DDF at 48–72 h (Supplementary Figure S1G) with an average of ∼4 DDF per cell (Figure 2A). Tin2DN stage 3 (120–168 h) showed cell accumulation in G1 with some cells remaining in G2; during this secondary proliferation arrest, S-phase cells decreased steadily as cells entered senescence (Figure 1B and C). End-of-stage-3 cell cycle distribution was consistent with observations for telomere-mediated senescence and for Tin2DN induction in normal cells (57,58,63).

To validate that the observed phenotype was not specific to HCA2 fibroblasts or to Tin2DN-mediated shelterin dysfunction, we used the same Dox-inducible strategy in normal BJ fibroblasts to deplete the shelterin components Tin2, TRF2 or POT1 via shRNA expression and achieved the same multistep senescence (Supplementary Figure S2). Thus, no matter how shelterin or telomeric chromatin was disrupted, the primary telomere-mediated proliferation arrest (stage 1) was transient in p53 wild-type cells and preceded reproliferation (stage 2) before entry into senescence (stage 3).

### TU causes non-telomeric DNA breaks and genomic instability

To further evaluate the effects of shelterin dysfunction on DDF dynamics during Tin2DN induction, we quantified 53BP1 foci (Figure 2A). Again, we observed three distinct stages. In the first 48 h (stage 1), cells showed a gradual increase of DDF consistent with TU induction, which plateaued between 48 and 96 h (stage 2). Then, concordant with reproliferation (Figure 1B and C), a secondary increase of DDF was observed (stages 2 and 3). p16^INK4a^ expression did not change the number of 53BP1 foci at any time (Figure 2A). Because Tin2DN was induced throughout the experiment, we speculated that the secondary DDF increase was not related to the primary TU caused by shelterin dysfunction. To directly evaluate telomere-associated DDF (i.e. TIF), we performed tFISH in combination with 53BP1 staining (Figure 2B). DDF of the first and second stages (0–96 h) colocalized with telomeres, validating TIF formation (2,16). However, the secondary increase of DDF (stages 2 and 3) overlapped with the reproliferation phenotype and no longer localized with telomeres, suggesting the formation of non-telomeric DSBs (non-TIF). We hypothesized that this secondary DDF resulted from division with dysfunctional telomeres, which could generate genomic instability as seen with TU in p53-deficient cells (1,33,36,37). Genomic instability as micronuclei, chromosome bridges and mitotic catastrophes was increased after 144 h of Tin2DN induction, coinciding with reproliferation (Figure 2C and Supplementary Figure S3A). Alternatively, no genomic instability was observed in p16^INK4a^-induced cells nor immediately after the primary Tin2DN-induced proliferative arrest for up to 96 h. Consistent with a phenotype of genomic instability, an increased duration of mitosis was observed in Tin2DN-induced cells containing H2B-GFP-labeled chromatin (Figure 2D and Supplementary Figure S3B).

While TIF are enriched in senescent cells, non-TIF can also constitute a significant fraction of detected DDF (18–20). We thus examined whether TIF or non-TIF were responsible for entry into telomere-mediated senescence. We induced Tin2DN expression for 120 h until the end of reproliferation and stopped the induction to remove TIF. Importantly, the integrated intensity of tFISH per nucleus remained unchanged, suggesting that telomeres were not directly damaged during the prolonged Tin2DN induction (Supplementary Figure S3C). This observation was supported using quantification of telomere length by terminal restriction fragment analysis using Southern blot (Supplementary Figure S3D). As expected, TIF removal resulted in a decrease in total DDF, while non-TIF remained unaffected (Figure 2E and F, and Supplementary Figure S3E). Strikingly, the cells did not restart DNA synthesis but developed SA-B-GAL (Figure 2G and Supplementary Figure S3F), suggesting that TIF were not required for senescence following non-TIF formation. Importantly, in cells with depleted Tin2, TRF2 and POT1, we reproduced the distinct phases of DDF formation with the second phase coinciding with genomic instability (Supplementary Figure S3G–J). Thus, TU is a multistep senescence trigger but is not itself required to maintain stable senescence.

### TU results in a weak and transient p53-dependent DDR

**Figure 3.**
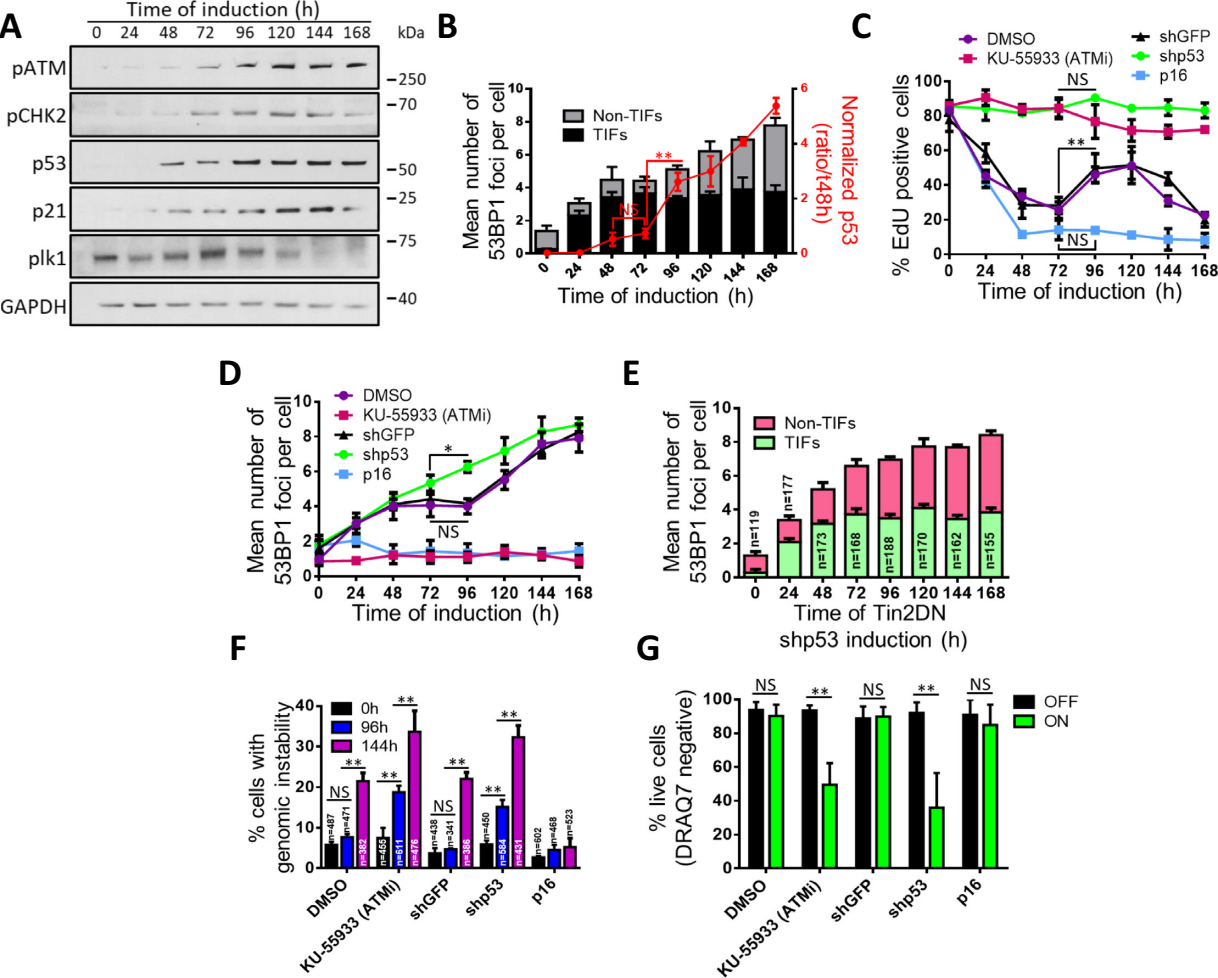
Primary telomere dysfunction induces a transient ATM/p53-dependent cell cycle arrest. (**A**, **B**) BJ cells expressing inducible Tin2DN were treated with Dox. (**A**) Whole cell lysates analyzed by western blot for DDR-activated proteins pATM (S1981-ATM) and pCHK2 (Thr68-Chk2) as well as p53, p21 and PLK1 (mitosis control). (**B**) Normalized p53/GAPDH ratio (red line) reported with the p53 signal at 48 h representing 1× fold (three independent experiments). In the background, the mean number of 53BP1 foci per nuclei is reported for experimentally matched cells and split into TIF (black) and non-TIF (gray). (**C**–**G**) HCA2 cells expressing inducible Tin2DN and shGFP or shp53, or only p16, were treated with Dox. Cells expressing only Tin2DN were treated with 10 μM of Ku-55933 (ATMi), or solvent (1:1000 DMSO). (**C**) Percentage of EdU-positive cells from 24-h EdU pulses. (**D**) Mean number of 53BP1 foci per nuclei. (**E**) Telomeres probed by tFISH for TIF or non-TIF in Tin2DN cells treated with shp53. Mean number of 53BP1 foci per nuclei is shown. Foci colocalized with telomeric probes (TIF, green) or not (non-TIF, pink). (**F**) Percentage of DAPI-stained cells showing genomic instability. (**G**) Following 72 h with (ON) or without (OFF) Tin2DN induction, cells were assessed for cell viability by DRAQ7 and flow cytometry. Percentage of live cells (DRAQ7 negative) is reported. All quantified data are the mean ± SD of triplicates and represent at least three independent experiments. NS: non-significant. Two-way ANOVA: *P< 0.05; **P< 0.01.

TU activates an ATM-dependent DDR that is defined by p53/p21 activation and weak CHK2 activity, which are all important to maintain the stability of the telomere-mediated senescence (2,23,24,42,64). To test whether TIF and non-TIF had a differential impact on DDR dynamics during multistep senescence, we first measured ATM activation by ATM-S1981 phosphorylation (Figure 3A). ATM activity gradually increased to a peak plateau consistent with the appearance of non-TIF and genomic instability at around 120 h post-induction (Figure 2B and C). Similarly, we did not observe any significant activation of CHK2 (phospho-T68) after the primary TU response (24–48 h), but detected increased phosphorylation after reproliferation (Figure 3A and C). These data were consistent with a weak, early stabilization of p53 and a weak increase of its downstream target, p21 (Figure 3A). Overall, the normalized levels of p53 to TIF/non-TIF data from Figure 2B reflected the overall DDR pattern where the appearance of non-TIF at 96–120 h of induction corresponded with a strong DDR (Figure 3B). In addition, the disappearance of PLK1 (65), an essential kinase for mitosis, indicated that cells were phasing out of the cell cycle (Figure 3A). Overall, we observed two waves of activation of the DDR cascade: the first wave is caused by TU and is unable to maintain cell cycle checkpoints; and the second was triggered by secondary non-TIF breaks and genomic instability, and provided stable arrest.

The weak activation of p53 during early Tin2DN induction is inconsistent with reports of TU establishing senescence via a direct DDR–p53 pathway (2,23). To validate the role of p53 during DDR wave 1 or 2, we depleted p53 using shRNA (shp53) (Supplementary Figure S4A). Tin2DN induction in shp53 cells did not show any DNA synthesis arrest (Figure 3C) but revealed a faster accumulation of DNA damage (Figure 3D) defined as non-TIF and genome instability (Figure 3E and F). This demonstrated that p53 was required for both primary and secondary TU-induced proliferative arrests. The importance of the DDR–p53 pathway was further demonstrated by inhibiting ATM (Supplementary Figure S4C and D), which must be activated for senescence (2,42). Similar to p53 depletion, ATM inhibition by the small molecule Ku-55933 prevented the primary proliferative arrest, but also prevented formation of 53BP1 DDF (an event downstream of ATM) and increased the genomic instability (Figure 3C, D and F). With blocked ATM–p53 cell cycle checkpoints, most Tin2DN-induced cells underwent mitotic catastrophe and cell death at around 72 h, as confirmed by FACS (Figure 3G and Supplementary Figure S4D). Tin2DN-induced shp53 cells also showed a dramatic increase in chromosome end-to-end fusions (Supplementary Figure S4E), which has been shown in p53-altered cells (1,36,37). Time-lapse experiments followed cell cycle progression in Tin2DN-induced shp53 cells and showed a high frequency of mitotic abnormalities (division of dicentric chromosomes) from the start of induction (Supplementary Figure S4F). Cells continued dividing while accumulating signs of damage, suggesting that mitosis induced genomic instability. Lengthy mitoses took over 3 h and resulted in mitotic catastrophe and nuclei with lagging chromosomes, initiating breakage–fusion–bridge cycles that are observed in cancer (66,67).

### Reproliferation leads to genome instability and non-TIF

**Figure 4.**
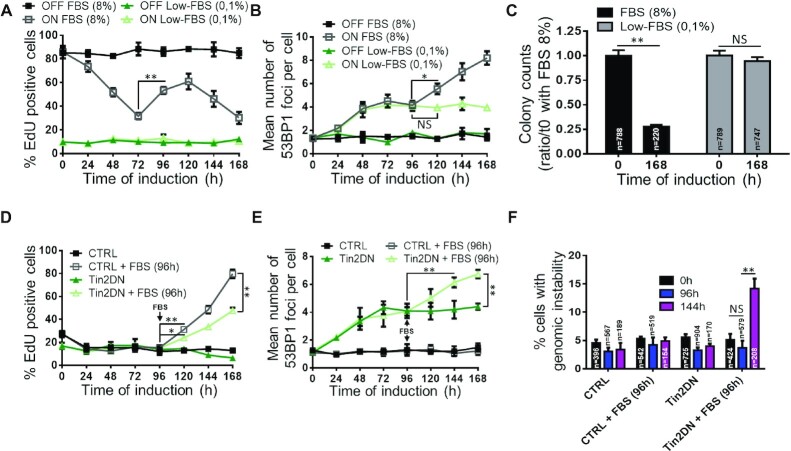
Non-TIF mediate telomeric senescence. (**A**–**C**) BJ cells expressing inducible Tin2DN were treated with (ON) or without (OFF) Dox and fixed daily. Cells were cultured in serum starvation medium (0.1% FBS) or in standard proliferative condition (8% FBS). (**A**) Percentage of EdU-positive cells from 24-h EdU pulses. (**B**) Cells stained for 53BP1 and DAPI to quantify mean number of 53BP1 foci per nuclei. (**C**) After 7 days of culture in normal or serum starvation medium, cells were plated under standard conditions for 5 days. Colony counts (clonogenic assay) are a ratio against time 0 with 8% FBS. (**D**–**F**) BJ cells expressing inducible Tin2DN or not (CTRL) were treated with Dox and fixed daily. Cells were cultured in serum starvation medium and, where indicated, FBS was added to the medium (up to 8%) after 96 h of Tin2DN induction. (**D**) Cells were treated with EdU as in (A). (**E**) Cells were stained as in (B) for 53BP1 foci. (**F**) Percentage of DAPI-stained cells showing genomic instability after Dox treatment. All quantified data are the mean ± SD of triplicates and represent at least two independent experiments. NS: non-significant. Two-way ANOVA: *P< 0.05; **P< 0.01.

To further test whether reproliferation and non-TIF are required for TU-induced senescence, we blocked cell proliferation via a G0/G1cell cycle arrest by using FBS starvation (68) (0.1% FBS; Figure 4A). Serum-starved Tin2DN-induced cells developed primary DDF at a similar rate to cells in normal conditions (8% FBS), but did not display a secondary DDF increase (Figure 4A and B). Moreover, serum starvation inhibited SA-B-GAL even after 7 days of TU (Supplementary Figure S5A), suggesting that the primary DDF from TU was not sufficient to induce senescence. To further validate the absence of senescence in low serum and the stability of senescence under normal serum, both conditions of Tin2DN-induced cells were released from Dox and normal serum was added at 168 h post-induction to score colony formation potential. Strikingly, low-serum cells completely recovered their proliferation capacity, demonstrating that TU was fully reversible. However, normal serum cells did not recover and confirmed that only TU combined to a reproliferation phenotype confers a stable irreversible senescence state (Figure 4C). Indeed, adding serum back to serum-starved Tin2DN-induced cells at 96 h after induction was sufficient to restimulate proliferation and increased the DDF (Figure 4D and E) and genome instability levels (Figure 4F). Thus, TU is an indirect, multistep senescence trigger in the context of continued cell proliferation but is not itself sufficient to establish or maintain stable senescence.

### Sister chromatid fusions associated with TU require homologous recombination

**Figure 5.**
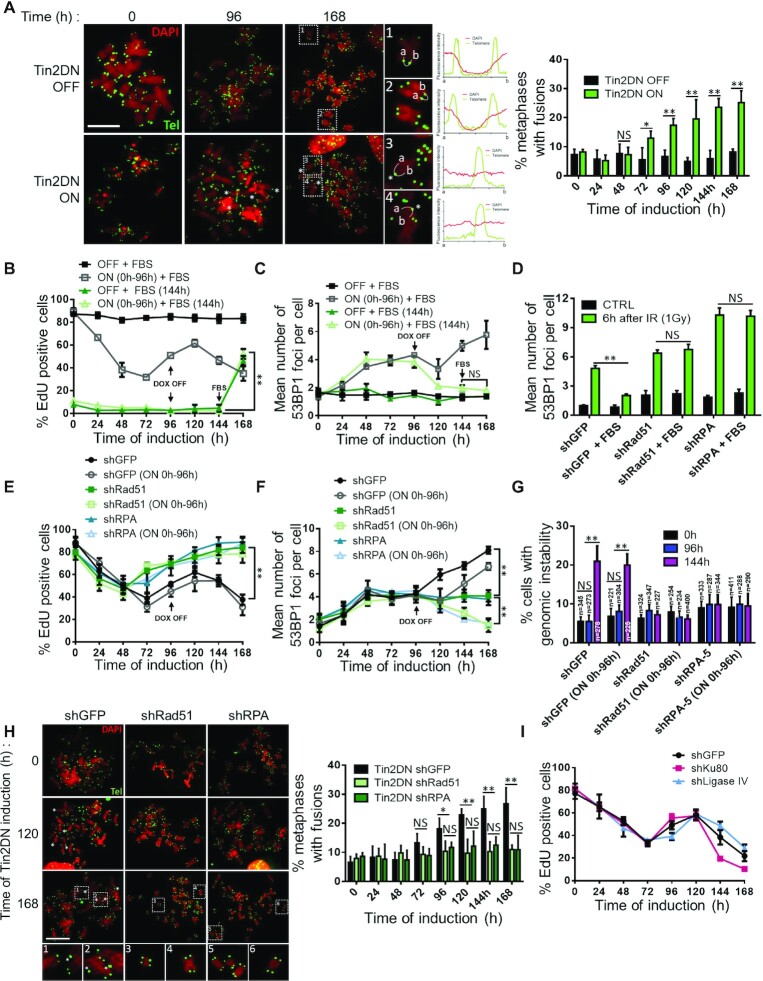
TU induces S-phase-dependent sister chromatid fusions mediated by homologous recombination (HR). (**A**) BJ cells expressing inducible Tin2DN were treated with (ON) or without (OFF) Dox and with 100 ng/ml of nocodazole 4 h prior to fixation. Cells were stained with DAPI (red), and telomeres were probed using tFISH (green). Asterisks denote sister chromatid fusions. The fluorescence intensity for DAPI and telomere was linearly assessed between a and b (curved white line in pictures labeled 1–4). The middle panels show representative telomere (green) and DAPI (red) signal spreading. The right panel shows the percentage of metaphases showing at least one sister chromatid fusion. Scale bar: 20 μm. (**B**, **C**) BJ cells expressing inducible Tin2DN were treated for 96 h with (ON) or without (OFF) Dox and fixed daily. Cells were cultured in standard proliferative condition (8% FBS; squares) or in serum starvation medium (0.1% FBS; triangles) for 144 h at which time FBS was added to the medium (up to 8%). (**B**) Cells were pulsed with EdU for 24 h. (**C**) Cells stained for 53BP1 and DAPI to quantify the mean number of 53BP1 foci per nuclei. (**D**–**G**) BJ cells expressing inducible Tin2DN and shGFP, shRad51 or shRPA. (**D**) Cells were irradiated with 1 Gy or untreated (CTRL) and fixed 6 h after treatment. Cells were stained for 53BP1 foci as in (C). (**E**, **F**) Where indicated (ON 0h-96h), Dox treatment lasted only for 96 h. (**E**) Cells were pulsed with EdU for 24 h. (**F**) Cells were stained for 53BP1 foci as in (C). (**G**) Percentage of DAPI-stained cells showing genomic instability. (**H**) Cells were treated and data analyzed as in (A); the left panel shows representative images, and the right panel displays the percentage of metaphases showing at least one sister chromatid fusion. Scale bar: 20 μm. (**I**) BJ cells expressing inducible Tin2DN and shGFP, shKu80 or shLigase IV. Cells were treated with Dox and pulsed with EdU for 24-h windows. All quantified data are the mean ± SD of triplicates and represent at least two independent experiments. NS: non-significant. Two-way ANOVA: *P< 0.05; **P< 0.01.

To analyze TU-induced genomic instability in normal cells, we examined metaphase chromosomes captured in mitosis during the reproliferation of p53 wild-type, Tin2DN-induced cells. We noticed a significant loss of dual tFISH telomeric probes, which indicated sister chromatid alterations (Figure 5A) and were similar to the chromosomal fusions described in cells escaping senescence (69,70) and to telomeric sister losses (24). Indeed, the presence of telomeric fusions during reproliferation after Tin2DN induction was directly visible as an increase in telomere length detected on Southern blot (Supplementary Figure S3D). These potential sister chromatid fusions or losses were the only notable chromosomal aberrations in normal cells, suggesting that the end-to-end fusions in p53-altered cells (Supplementary Figure S4E) (1,34–37) did not occur or occurred at very low frequency and prevented passage into M phase (71). In addition, if fusions had occurred during G1 phase between non-replicated chromosomes, we would have observed dicentric chromosomes after DNA replication (52).

Since DSB repair is inhibited during mitosis (72), we hypothesized that telomeric fusions occurred during late S/G2 phases, when two sister chromatids would be available (24). To validate our hypothesis, we used additional TU inducers (Tin2, TRF2 or POT1 depletion), and in all cases, sister chromatid fusions were dominant (Supplementary Figure S5B). To confirm the importance of DNA synthesis and cell cycle phases in the sister chromatid fusion phenotype, we examined whether serum starvation prevented fusions by suspending Tin2DN-induced cells in G1/G0. Cells in normal serum were induced for 96 h until just after reproliferation, and Dox was removed (Figure 5B, empty squares). Induction for 96 h was sufficient to promote a secondary DDF as observed during continued Tin2DN expression (Figure 5C, empty squares). Alternatively, removing Dox at 96 h in serum-starved cells resulted in DDF diminishing due to TU resolution (Figure 5C, empty triangles). We added serum back to starved cells at 144 h, which fully restored proliferation (Figure 5B, empty triangles) but did not induce secondary DDF (Figure 5C, empty triangles). Hence, our results suggest that DNA synthesis in the presence of TU is necessary to induce secondary DDF and that DNA template-dependent sister chromatid fusions are involved, rather than fusions between different chromosome ends. This is also consistent with results obtained using a TRF2 mutant where contact inhibition prevented fusions between chromosomes (52).

Chromosome end fusions can result from the DSB repair pathways, HR or NHEJ. HR requires a homologous DNA template and is only possible in S/G2 phases (73). This stage coincides with the disappearance of dual sister chromatid signals at chromosome ends. We hypothesized that HR is required for secondary DSBs that result from TU. We depleted key HR proteins (Rad51 and RPA) (74) using shRNA in Tin2DN-inducible cells (Supplementary Figure S5C) and validated DSB repair kinetics in HR-depleted, non-induced Tin2DN cells exposed to X-ray irradiation. These cells showed reduced DNA repair when compared to control shGFP cells and showed no differences in repair kinetics with or without serum, confirming that NHEJ was the residual cell cycle-independent repair mechanism (Figure 5D). We then induced Tin2DN continuously or for a 96-h pulse until reproliferation in normal cells (shGFP) and followed DNA synthesis and DDF accumulation over time. First, HR defects (shRPA and shRad51) did not affect the primary proliferation arrest or DDF induced by TU, suggesting that the damage response originating from TU does not depend on HR (Figure 5E and F). However, HR defects eliminated the secondary proliferative arrest when Tin2DN was continuous or pulsed (Figure 5E). Importantly, when induction was stopped at 96 h, HR wild-type shGFP cells accumulated DDF from secondary DSBs, whereas HR-depleted cells rapidly returned to base levels of DNA damages/foci, suggesting that there was no telomeric fusion event (Figure 5E). Genomic instability occurred only in HR wild-type cells and was consistent with the absence of sister chromatid fusions in HR-depleted cells (Figure 5G and H).

To further assess the importance of HR and eliminate the possibility of end-to-end telomeric fusions, we depleted essential NHEJ DNA repair components (Ku80 and Ligase IV; Supplementary Figure S5D) (75,76). We again used irradiation with or without serum to validate DNA repair kinetics and observed that NHEJ-depleted cells were greatly affected in the absence of serum when HR was not available for repair (Supplementary Figure S5E). Impressively, NHEJ depletion did not have an impact on primary/secondary proliferative arrests (Figure 5I) or on DDF accrual and genome instability (Supplementary Figure S5F and G). To determine whether HR-mediated sister chromatid fusions created *de novo* DNA lesions in senescing cells, we performed a neutral comet assay, which detects genomic DSBs (77). Therefore, primary TU itself should not be detected in this assay. As expected, 72 h of Tin2DN induction did not generate any comets, but secondary DNA lesions at 168 h did (Supplementary Figure S5H). Again, when HR was blocked, no DNA fragments were detected, whereas NHEJ had no impact on comet formation. These data are concordant with non-TIF, which appear only under conditions that give rise to comets (Supplementary Figure S6). Thus, the DNA repair mechanism of HR, but not NHEJ, mediates TU-derived sister chromatid fusions that induce genomic instability, or TU-derived genome instability in normal senescing cells.

### Natural replicative senescence is established by genomic instability derived from TU

**Figure 6.**
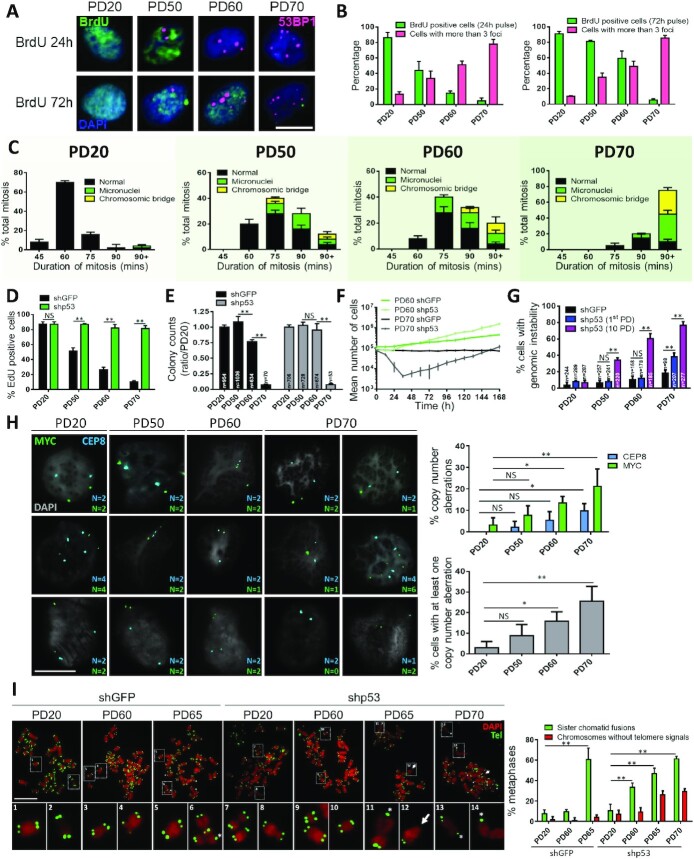
Natural replicative senescence requires genomic instability. Cells were grouped in four populations (PD20, PD50, PD60 and PD70). (**A**, **B**) Cells were treated with BrdU 24 or 72 h prior to fixation. (**A**) Cells were stained for BrdU (green), 53BP1 (red) and DAPI (blue). Scale bar: 20 μm. (**B**) Percentage of BrdU-positive cells for 24-h (left panel) and 72-h (right panel) pulses, associated with the percentage of cells with >3 foci. (**C**) Cells infected with H2B-GFP lentivirus were cultured for live cell imaging (IncuCyte). Duration of complete mitosis is shown for the different PDs and abnormal events are reported for each PD. (**D**–**G**) BJ cells were infected to express shGFP or shp53. (**D**) Cells were cultured for 2 days prior to sampling. Percentage of EdU-positive cells from 24-h EdU pulses. (**E**) Cells were replated under standard proliferative conditions (8% FBS) allowing proliferation for 5 days. Colony counts (clonogenic assay) are reported as a ratio to PD20 shGFP counts. (**F**) Cells counts at every 12 h for PD60 and PD70 immediately following lentiviral infections. (**G**) Percentage of DAPI-stained cells showing genomic instability 48 h after infection (equating to 1st PD with shGFP and shp53) or for 10 PDs with shp53 after lentiviral infection. (**H**) BJ cells at the indicated PD were probed using FISH for the CEP8 (blue) and MYC (green) genes and nuclei were stained using DAPI (gray). Left panel shows representative images of the quantified CEP8 and MYC probes (right graphs). Copy number aberration was indicated when the probe count was not 2 (G1) or 4 (S–G2–M). Aberrations for each probe (top graph) and aberrations per cell (at least one probe count is not 2 or 4, or they are not the same; bottom graph). Scale bar: 20 μm. (**I**) BJ cells were infected to express shGFP or shp53 for 48 h and were treated with 100 ng/ml of nocodazole 4 h prior to fixation. Cells were stained with DAPI (red), and telomeres were probed using tFISH (green). Asterisks denote sister chromatid fusions and arrows mark chromosome breaks. Panel shows the percentage of metaphase with at least one fusion (green) or one chromosome without telomeric signals (red). Scale bar: 20 μm. All quantified data are the mean ± SD of triplicates and represent at least two independent experiments. NS: non-significant. Two-way ANOVA: *P< 0.05; **P< 0.01.

To confirm that the natural attrition of telomeres applies the same mechanisms we observed with acute TU, we cultured normal BJ cells to senescence and isolated five representative cell populations at increasing PD (PD20, PD40, PD50, PD60 and PD70; Supplementary Figure S7A). Similar to earlier studies (78), these populations had distinct proliferative profiles with doubling times ranging from 36 h for early passage cells (PD20, PD40) to >200 h at PD70, which were senescent (Supplementary Figure S7B and C). Most PD70 cells did not proliferate, displayed the highest SA-B-GAL and the lowest tFISH total intensity (short telomeres), and generated the most DNA damage comets (Supplementary Figures S3C and S7D and E). As PD increased, 53BP1 DDF confirmed the presence of critically short telomeres leading to TIF (2,4,31). This correlated with a decrease in DNA synthesis, based on short (24 h) or long (72 h) BrdU pulses (Figure 6A). Short/long BrdU pulses showed that PD50 and PD60 populations (pre-senescence) contained an increasing proportion of cells (∼40% at PD60) with slower cell cycles that required longer pulses to reveal DNA synthesis (Figure 6B). Single-cell analysis (Supplementary Figure S8A and B), particularly within the PD60 population, showed that cells tolerated dysfunctional telomeres and continued to divide with TIF as previously suggested (28–31), albeit at reduced rates (∼95% DDF positive and 60% 72-h BrdU positive). A time lapse of H2B-GFP chromatin in live cells showed that increasing PDs increased mitotic transit time and micronuclei or chromatin bridge formation (Figure 6C). This suggests that dysfunctional telomeres were tolerated and accumulated during the natural replicative life span, slowing the cell cycle and sporadically triggering TU-derived genomic instability in a proportion of cells.

To test this idea, we acutely depleted p53 (shp53) to restart proliferation in near-senescent or senescent cells (23). Senescent PD70 cells and advanced passage cells showed immediate DNA synthesis after p53 depletion, confirming that TU accumulation slows the cell cycle via a transient p53-mediated checkpoint (Figure 6D). Longer evaluation using clonogenic assays revealed that shp53 weakly restored long-term proliferation in PD70 cells (Figure 6E), suggesting that these cells were already genetically unstable and could not readily multiply. Accordingly, cell numbers did not immediately increase in PD70-shp53 cells as they did in PD60-shp53 cells (Figure 6F). Instead, cells entered mitotic catastrophe and died (Supplementary Figure S8C and D). As with previous reports of senescence emergence (23), PD70-shp53 cells eventually proliferated from a clonal emergence of ∼5% of cells (colony assay, Figure 6E). Thus, we hypothesized that restarting proliferation after senescence from TU-derived genomic instability led to more DNA perturbations and mitotic crisis in most cells. Two days following p53 depletion (enough for one division), genomic instability was significantly increased only in senescent PD70 cells (Figure 6G; 1st PD), demonstrating that only this cell population had prior TU-derived genome instability. Alternatively, p53-depleted PD50–60 cells required multiple PDs to develop instability and suggested that genome instability was not yet prevalent in pre-senescent populations (Figure 6G; 10 PD). To further demonstrate that genomic instability occurs during natural senescence and is more prevalent in terminally senescent cells, we used FISH to detect chromosomal abnormalities. Alterations in interphase copy number (not *n* = 2 in G1 or *n* = 4 in G2) were highly correlated with increasing PDs and thus telomere dysfunction (Figure 6H).

Finally, metaphase and anaphase chromosomes captured in the first mitosis of PD70-shp53 cells behaved exactly as observed with Tin2DN-induced cells: predominance of sister chromatid fusions as revealed by tFISH and no detectable end-to-end fusions (Figure 6I). Notably, chromosomes without telomere signals were found in p53-depleted PD65–70 cells but not in the PD65-shGFP controls, reinforcing the idea that pre-senescent, p53 wild-type cells have not yet acquired genomic instability required for senescence. Since nearly all PD60–70 cells harbor DDF and telomeres short enough for fusions (Figure 6B and Supplementary Figure S8A and B), the incidence of sister chromatid fusions observed in mitotic PD65-shGFP cells (∼60% of mitoses; Figure 6I) suggests that only this proportion of cells will senesce following mitosis exit, leaving other cells to attempt additional divisions. This agrees with the diminishing long-term division capacity (colony formation) between PD60 and PD70 as the cell population enters terminal replicative senescence (Figure 6E).

## DISCUSSION

**Figure 7.**
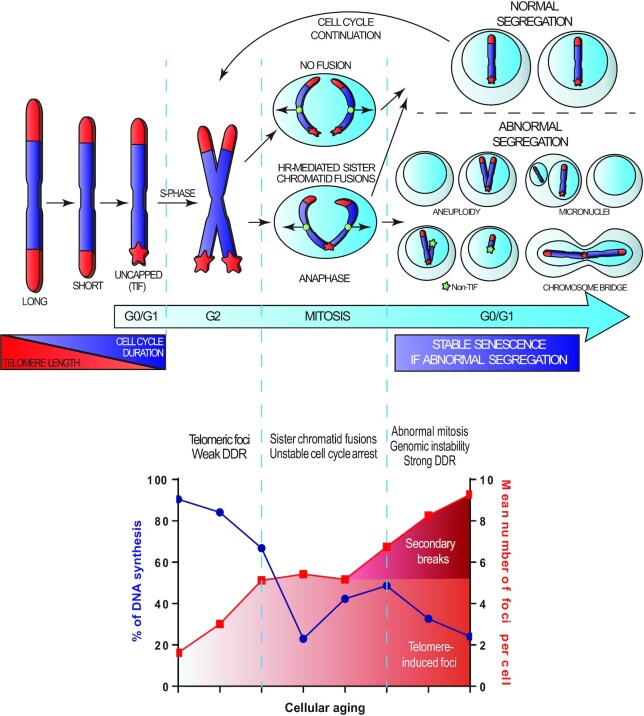
A multistep model defining entry into telomere-mediated senescence. The top panel highlights that telomere shortening leads to uncapping and the apparition of TIF (red star). Cells with TU are susceptible for sporadic sister chromatid fusions mediated by HR. As cells sustain and tolerate TIF, additional divisions with dysfunctional and potentially fused telomeres lead to abnormal chromosomal segregation, genomic instability and random secondary non-TIF DSBs (green star), which are the true inducers of a strong DDR and a stable senescence-associated proliferation arrest. The bottom panel represent an alignment between the time course of EdU proliferation and the timeline of 53BP1 DDF formation. We conclude that telomere-induced senescence initiates with a weak and transient DDR via TIF formation eventually followed by a stable senescence arrest associated with secondary non-telomeric (non-TIF) DNA lesions.

The current telomere-mediated senescence model proposes that irreparable TIF are responsible for stable senescence-associated proliferation arrest by activating a telomere-centric DDR that sustains chronic p53 activation (2,18–21,79). Our study proposes an updated model involving a multistep entry into senescence (Figure 7). We propose that TIF act as indirect triggers with two main functions: (i) they provide a weak DDR to slow the cell cycle in cells with accumulating dysfunctional telomeres and (ii) they provide a substrate for HR-mediated, sporadic, telomeric sister chromatid fusions. In our model, the molecular cascade leading to stable cell cycle arrest and senescence is dependent on HR-mediated secondary genomic instability that follows sister chromatid fusions. This new model is consistent with multiple reports describing normal cells with a tolerance for TIF before entering senescence (28,29,31) and agrees with the notion that TIF often represent irreparable telomeric DSBs (18–20,79).

To explain the secondary genomic instability, we showed that the DDR induced by primary telomere dysfunction was weaker than that induced by genomic instability, which allows bypass of the primary TU-induced cell cycle arrest in the presence of a mitogenic stimuli like serum. Interestingly, during natural senescence, relatively old PD60 cells displayed two or more TIF per cell (Supplementary Figure S8B), yet they rarely underwent senescence in subsequent mitoses, as they still formed colonies (∼75%, Figure 6E). This suggests that TU-associated sister chromatid fusions are a relatively rare sporadic event. Given that telomeric sister chromatid fusions in normal cells required both S phase and HR in the presence of pro-mitotic signals (serum), we conclude that most of the time only cells undergoing mitosis with multiple TU can fully reach a stable, irreversible senescence. In this context, true telomere-mediated senescence is possibly a relatively rare event *in vivo* and earlier identification of resident senescent cells in tissue that used TIF as a single marker may have overestimated their occurrence (80). Notwithstanding their rare occurrence, these truly senescent cells may have been diluted by competition with growing cells or by potential clearance, which could explain their low abundance and the difficulties in identifying these cells (81,82).

Previous work has shown that TU in p53-defective cells results in NHEJ-mediated chromosomal end-to-end fusions (1,37,83) followed by breakage–fusion–bridge cycles that promote genomic instability observed in cancer (66,67). Given that our controlled activation of TU in normal cells still allowed for division in the presence of weak DDR signaling without NHEJ-mediated end-to-end fusions, our data suggest that p53 inhibits telomeric NHEJ-mediated end-to-end fusions but cannot prevent sporadic HR-mediated telomeric sister chromatid fusions that also lead to genomic instability and subsequent p53-driven senescence. Of note, a recent study reveals that the shelterin POT1 suppresses HR-mediated repair at telomeres in HEK293E cells supporting a model similar to ours where HR can cause dysfunctional telomere fusions (84). Overall, this suggests that both NHEJ (end-to-end) and HR (sister chromatids) can contribute to telomeric fusions in context-dependent manners. For sister chromatids, it is possible that the HR-mediated telomeric fusions are generated during late S-phase DNA replication as non-covalently fused replication intermediates (85). Interestingly, this is also consistent with a proposed three-state TU model (41) as the strategies used in our study for TU could place the telomeres in their intermediate state, when NHEJ-mediated end-to-end fusions are inhibited (42). Alternatively, in the absence of p53, continual proliferation further erodes the telomeres to allow their state of complete opening and the end-to-end fusions associated with replicative crisis occurs, which in addition to generating genome instability can also contribute to tumor suppression via induction of cell death (21). In normal cells, our results are also consistent with previous observations that TRF2 mutants can disrupt shelterin in fibroblasts with wild-type p53 to generate genomic instability (51,52). However, in one of these studies (52), TFR2 disruption yielded NHEJ-mediated end-to-end telomeric fusions between different telomeres, which we did not observe in any of our TU strategies, including normal senescence. Interestingly, despite high levels of end-to-end fusions, telomeric sister chromatid fusions were observed at a significant level (52). Given the potential context dependence of an NHEJ–HR choice for telomeric fusions, perhaps we can suggest that the specific TRF2 mutant that was used can favor NHEJ end-to-end telomeric fusions creating a mixed fusion phenotype in p53 wild-type cells. Overall, we show that the appearance of telomeric fusions in normal cells requires chromosomal condensation in M phase and was only detected in the very last division of p53 wild-type cells entering senescence. This may explain why our observations were never clearly reported before as a key senescence trigger in normal cells.

In conclusion, we demonstrate that replicative senescence, a tumor suppressor mechanism and guardian of genome stability, sometimes requires genomic instability to initiate its own action. Cell crisis was previously proposed to unite genome instability to tumor suppression; our data would suggest that senescence paradoxically also works in a similar manner (21). Along those lines, a high frequency of polyploidy and multiple chromosomal abnormities have been recently reported in cells approaching senescence in distinct studies (43–48). Others have shown that forced polyploidy can induce senescence (47,48) or reported that replicative senescence can induce aneuploidy and whole chromosome instability (47,49,50). This agrees with the telomere-induced senescence model that we propose, which reconciles our observations of senescence-associated genomic instability with two notions: (i) telomere breaks are mostly irreparable and (ii) cells can tolerate TIF during an unstable ‘pre-senescent’ state. This new model for entry into telomere-mediated senescence offers a new basis for stress- or age-associated genome damage, but also suggests that cells escaping telomere-mediated senescence almost automatically harbor irreparable genome lesions. Importantly, this model suggests that strategies targeted at repairing telomeres in pre-senescent cells could simultaneously eradicate TIF and low-level DDR signaling, while preventing further irreversible genome lesions.

## DATA AVAILABILITY

The nucleotide sequences of all synthetic nucleic acid fragments used in this study, including details of PCR primers, are available in the Supplementary Data.

## Supplementary Material

gkab965_Supplemental_File
