# Supplementary material for: Homologous recombination-mediated irreversible genome damage underlies telomere-induced senescence
Source: Nucleic Acids Res. 2021 Nov 2;49(20):11690–707. doi: 10.1093/nar/gkab965 (PMC8599762; doi:10.1093/nar/gkab965)
Supplement: gkab965_Supplemental_File [file gkab965_supplemental_file.pdf]

Supplemental figure legends

Figure S1

(A) Schematic representation of the Tin2DN construct. The CMV promoter is controlled by a Tetracycline operator (TetO) for expression of Tin2DN and reporter gene eGFP, contained in an IRES bicistronic vector. **(B)** HCA2 fibroblasts expressing inducible Tin2DN treated with (+) or without (-) Dox for 48 h. Western blot of whole cell lysates for Tin2, GFP and GAPDH (housekeeping) proteins. **(C-G)** HCA2 expressing inducible Tin2DN, p16 or TetR alone (CTRL) treated with Dox. **(C)** Merged immunofluorescence images of representative cells at each time point, taken with a DAPI (blue, nuclear counterstain) or a FITC (for GFP emission) filter. Scale bar: 20 µm. **(D)** Representative Tin2DN-induced stained cells for γH2AX (green), 53BP1 (red) and DAPI (blue). Graph shows percentage of cells showing more than three γH2AX and 53BP1 positive foci after 96 h induction. Scale bar: 20 µm. **(E)** Graph shows percentage of SA-B-GAL positive cells represented in left panel. Scale bar: 200 µm. **(F)** Representative images of HCA2 cells expressing histone H2B-GFP and cultured in the live cell imaging system (Incucyte). Scale bar: 200 µm. **(G)** Number of DDF per single cells after indicated time of Tin2DN induction. After 48 h of induction, almost all cells present at least one foci. All quantified data are the mean ± S.D of triplicates and represent at least three independent experiments. NS: non-significant. Two-way ANOVA: * $p<0.05$; ** $p<0.01$.

Figure S2

BJ cells expressing inducible shGFP (control), shTin2, shTRF2 or shPOT1 were treated with Dox. **(A, B)** Percentage of EdU positive cells from 24 h EdU pulses. **(C)** After 48 h with (+) or without (-) Dox, whole cell lysates were analyzed by western blotting for Tin2 and GAPDH proteins. **(D)** After 48 h Dox induction (ON) or without (OFF), extracted RNA was reverse-transcribed for Sybr qPCR using TRF2 and POT1 primers. Results are compared to the control shGFP (no induction; OFF) and presented in a log2 scale. **(E)** Cells were fixed and assessed for SA-B-GAL. Scale bar: 200 µm. All quantified data are the mean ± S.D of triplicates and represent at least three independent experiments. Two-way ANOVA: ** $p<0.01$.

Figure S3

BJ cells expressing inducible Tin2DN, shGFP, shTin2, shTRF2 or shPOT1 were treated with Dox. **(A)** DAPI stained cells showing genomic instability (mitotic catastrophes, chromosome bridges or micronuclei) after 144 h of Tin2DN induction; white arrows point out aberrations. Scale bar: 20 µm. **(B)** Cells expressing H2B-GFP cultured for live cell imaging (Incucyte). Normal mitosis is shown (32h30) preceding two latent metaphases (108h and 162h15) leading to genomic instability as chromosome bridges and micronuclei, respectively. Three different cells are shown (1st, 2nd and 3rd). NEB: Nuclear Envelope Breakdown. ANA: Anaphase. TELO: Telophase. Scale bar: 20 µm. **(C)** Representative images of cells stained for DAPI and telomeres probed using tFISH (green). For the indicated PDs or indicated induction, the mean telomeric fluorescence intensity per cells was measured and reported in the graph. Scale bar: 20 µm. **(D)** HCA2 cells were cultured to replicative senescence (young versus old left panels) or Tin2DN was induced with Dox for up to 7 d (right panels). Representative southern blot of telomeres restriction fragments length assays is presented with DNA molecular weight labels. Telomere length distributions was quantified in the gel lanes and is presented graphically. bp : base pair. **(E-H)** Tin2DN was induced with Dox for only 120 h (OFF 120 h) or sustained throughout the experiment. **(E)** Representative images of cells

stained for 53BP1 (red) and DAPI (blue), and telomeres probed using tFISH (green). Co-localisation of probes is defined as a TIF. Fluorescence intensity for the signal of 53BP1 and the telomere was linearly assessed between a and b. Scale bar: 10 µm. **(F)** Graph shows the percentage of SA-B-GAL positive cells represented in panel. Scale bar: 200 µm. **(G, I)** Cells were stained for 53BP1 and DAPI. Mean number of 53BP1 foci per nuclei is shown. **(H, J)** Percentage of DAPI stained cells showing genomic instability. All quantified data are the mean ± S.D of triplicates and represent at least three independent experiments. NS: non-significant. Two-way ANOVA: ** p<0.01.

Figure S4

(A-E) HCA2 cells expressing inducible Tin2DN and shGFP or shp53, or only with inducible p16. **(B)** Following 4 h with (+) or without (-) 4 Gy of X-ray radiation and treatment with Ku-55933 (ATMi), cells were lysed. **(C)** Following 4 h with (+) or without (-) the indicated radiation or treatment duration with Dox, cells were lysed. **(C-D)** Cells expressing only Tin2DN were treated with 10 µM of Ku-55933 (+), or solvent (1:1000 DMSO) (-). **(A-C)** Western blots of whole cell lysates for p53 and GAPDH (housekeeping), and for **(B-C)** DDR-activated proteins pATM (S1981-ATM) and pCHK2 (Thr68-Chk2) as well as ATM and CHK2 proteins. **(D)** Following 72 h with (ON) or without (OFF) induction, cells were assessed for cell viability by DRAQ7 and flow cytometry. Gates were defined to include all the dead cells (DRAQ7 positive) which were not debris. **(E)** Cells were treated 72 h with Dox (ON/OFF) and with 100 ng/mL of nocodazole 4 h prior to fixation. Suspended cells were spread on glass slides, stained with DAPI (red) and telomeres were probed using tFISH (green). Graph shows percentage of cells in metaphase showing at least one end-to-end fusion. Panel shows representative cells with indicated double centromeres (white arrowheads). Scale bar: 5 µm. **(F)** Cells expressing H2B-GFP and shp53 were cultured for live cell imaging (Incucyte) during Tin2DN induction. Individual cells undergoing defective mitosis after Tin2DN induction (two different cells; 1st and 2nd). NEB: Nuclear Envelope Breakdown. ANA: Anaphase. TELO: Telophase. Right panel shows duration of each mitosis observed at single cell level, resulting in a linear correlation. Circular diagrams indicate percentage of abnormal mitosis detected. Scale bar: 20 µm. All quantified data are the mean ± S.D of triplicates and represent at least three independent experiments. NS: non-significant. Two-way ANOVA: ** p<0.01.

Figure S5

(A) BJ cells expressing inducible Tin2DN were cultured in serum starvation media (0.1% FBS) or in standard proliferative condition (8% FBS), and treated with Dox. At indicated times, cells were assessed for SA-B-GAL. **(B)** BJ cells expressing inducible shGFP, shTin2, shTRF2 or shPOT1 were treated 120 h with Dox (ON/OFF) and with 100 ng/mL of nocodazole 4 h prior to fixation. Panel shows representative cell spreading. Cells were stained with DAPI (red) and telomeres were probed using tFISH (green). Asterisks denote sister-chromatid fusions. Scale bar: 20 µm. **(C-H)** BJ cells expressing inducible Tin2DN and shGFP, shRad51, shRPA, shKu80 or shLigase IV. **(C, D)** Western blot of whole cell lysates for Rad51, RPA, Ku80, Ligase IV and GAPDH (housekeeping) proteins. **(E)** Cells were irradiated with 1 Gy dose and fixed 6 h after treatment. **(E, F)** Cells were stained for 53BP1 and DAPI to quantify the mean number of 53BP1 foci per nuclei. **(F, G)** Cells were treated with Dox and fixed at indicated time points. **(G)** Percentage of DAPI stained cells showing genomic instability at indicating time points. **(H)** At indicated times, cells were assessed for neutral comet assay (detection of DSBs). Graph shows the percentage of comet tail DNA represented in panel. Scale bar: 200 µm. All quantified data are the mean ± S.D of

triplicates and represent at least two independent experiments. NS: non-significant. Two-way ANOVA: * $p<0.05$; ** $p<0.01$.

Figure S6

(A) BJ cells expressing inducible Tin2DN and shGFP, shRad51, shRPA, shKu80 or shLigase IV. Representative BJ cells during Tin2DN induction are shown (top panels). Cells stained for 53BP1 and DAPI were quantified for mean number of 53BP1 foci per nuclei (bottom panel). Telomeres were probed using tFISH (green). Foci that co-localised with telomeric probes were TIF and those that did not were non-TIF. Scale bar: 20 µm. All quantified data are the mean ± S.D of triplicates and represent at least two independent experiments. NS: non-significant. Two-way ANOVA: ** $p<0.01$.

Figure S7

(A, B) BJ cells were continuously cultured in DMEM media containing 8% FBS. PD and doubling time (DT) were assessed for five distinct populations, resulting in a final selection of four different populations (PD20, PD50, PD60 and PD70) with different SA signatures. **(C)** Cells were cultured for live cell imaging (Incucyte) to determine the proliferative rate via cell confluence reported against time 0. (**D**) For indicated PDs, cells were assessed for SA-B-GAL. Percentage of positive cells (divided into low or high expression categories) is reported. **(E)** For indicated PDs, cells were assessed for neutral comet assay (detection of DSBs). Graph shows percentage of comet tail DNA represented in panel. Scale bar: 200 µm. All quantified data are the mean ± S.D of triplicates and represent at least two independent experiments. NS: non-significant. Two-way ANOVA: * $p<0.05$; ** $p<0.01$.

Figure S8

(**A-B**) Cells were treated with 10 µM of BrdU 24 or 72 h prior to fixation. Genomic double-stranded DNA was stained for BrdU, 53BP1 and DAPI. At indicated amounts of foci, percentage of BrdU positive and negative cells was assessed at each PD. **(C-D)** BJ cells expressing shGFP or shp53 were cultured for 2 days prior to experiments. Cell viability was assessed by DRAQ7 and flow cytometry. **(C)** Representative flow cytometry panels in which gates were defined to include all dead cells (DRAQ7 positive) that were not debris. (**D**) Percentage of live cells (DRAQ7 negative) is reported for each PD. All quantified data are the mean ± S.D of triplicates and represent at least three independent experiments. NS: non-significant. Two-way ANOVA: ** $p<0.01$.

Supplemental information

Supplementary Table 1. shRNA target sequences

Target gene symbol	Description	Sequences*
***RAD51* (Rad51)**	Rad51 recombinase	5’-TAGTCTGTTCTGTAAAGGGCG-3’
***RPA1* (RPA)**	Replication protein A1	5’-TTCCCAATCTTTAATCCAACG-3’
***TP53* (p53)**	Tumor protein p53	5’-GACTCCAGTGGTAATCTACT-3’
***XRCC5* (Ku80)**	X-ray repair cross complementing	5’-TTACTCATGGTAAAGCCCACG-3’
***LIG4* (Ligase IV)**	DNA ligase 4	5’-TAAAGGTGTTTAGATGCGAGC-3’
***gfp* (GFP)**	Green fluorescent protein	5’-GCTGGAGTACAACTACAAC-3’
***TINF2* (Tin2)**	TERF1 interacting nuclear factor 2	5’-GTGCAGCTCCGTCATTACCAT-3’
***TERF2* (TRF2)**	Telomeric repeat binding factor 2	5’-GCGCATGACAATAAGCAGA-3’
***POT1* (POT1)**	Protection of telomeres 1	5’-GGGTGGTACAATTGTCAATGT-3’

*Mature antisense are listed

Supplementary Table 2. Antibodies and probes

Antibodies	Source	Identifier	Dilution
**Rabbit anti-53BP1**	Novus Biologicals	Cat#NB100-305	1:2000
**Mouse anti-H2AX (S139; γH2AX)**	Millipore	Cat#JBW301	1:1000
**Mouse anti-p53**	Santa Cruz	Cat#sc-126	1:1000
**Rabbit anti-GAPDH**	Cell Signaling	Cat#2118S	1:2000
**Rabbit anti-pChk2 (Thr68)**	Cell Signaling	Cat#2661S	1:500
**Rabbit anti-Chk2**	Cell Signaling	Cat#2662	1:500
**Mouse anti-pATM (S1981)**	Cell Signaling	Cat#4526S	1:2000
**Mouse anti-ATM**	Abcam	Cat#ab78	1:2000
**Mouse anti-Tin2**	Novus Biologicals	Cat#NB600-1522	1:500
**Mouse anti-p21**	BD Biosciences	Cat#556430	1:1000
**Rabbit anti-GFP**	Abcam	Cat#ab290	1:1000
**Mouse anti-Rad51**	Abcam	Cat#ab213	1:1000
**Mouse anti-Plk1**	Abcam	Cat#ab17056	1:1000
**Rabbit anti-Ligase IV**	Santa Cruz	Cat#sc-28232	1:1000
**Rabbit anti-Ku80**	Cell Signaling	Cat#2180S	1:1000
**Mouse anti-RPA70**	Calbiochem	Cat#NA13	1:1000
**Mouse anti-BrdU**	BD Biosciences	Cat#347580	1:1000
**Donkey anti-mouse IgG (H+L) Cross-Adsorbed Secondary Antibody, Alexa Fluor 488**	ThermoFisher	Cat#A21202	1:800
**Donkey anti-rabbit IgG (H+L) Cross-Adsorbed Secondary Antibody, Alexa Fluor 488**	ThermoFisher	Cat#A21206	1:800
**Donkey anti-mouse IgG (H+L) Cross-Adsorbed Secondary Antibody, Alexa Fluor 647**	ThermoFisher	Cat#A31571	1:800
**Donkey anti-rabbit IgG (H+L) Cross-Adsorbed Secondary Antibody, Alexa Fluor 647**	ThermoFisher	Cat#A31573	1:800
**Goat anti-mouse IgG HRP-conjugated Secondary Antibodies**	Santa Cruz	Cat#sc-2005	1:10 000
**Goat anti-rabbit IgG HRP-conjugated Secondary Antibodies**	Santa Cruz	Cat#sc-2030	1:10 000
**Alexa Fluor 647 Azide, Triethylammonium Salt**	ThermoFisher	Cat#A10277	1:10 000
**Vysis CEP 8 SpectrumAqua Probe**	Abbott	Cat#06J54-018	1:50
**Vysis LSI MYC SpectrumGreen Probe**	Abbott	Cat#04N36-020	1:50

**TelC-Cy3 telomere probe, C-rich/leading strand**	Panagene	Cat#F1002	1:50

Supplementary Table 3. Sybr RT-qPCR primer sequences

Gene symbol	Sense	Description	Sequences
TERF2 **(TRF2)**	Forward	Telomeric repeat binding factor 2	5’-CAAGGAAGCTGCTGTCATTA-3’
TERF2 **(TRF2)**	Reverse	Telomeric repeat binding factor 2	5’-CCAGGAAGCGCAGCATCTTC-3’
POT1 **(POT1)**	Forward	Protection of telomeres 1	5’-AAATTGATGCATATCCGTGGT-3’
POT1 **(POT1)**	Reverse	Protection of telomeres 1	5’-CCCATACCCATGCTAACATCA-3’
TBP **(TBP)**	Forward	TATA-box binding protein	5’-GAGCCAAGAGTGAAGAACAG-3’
TBP **(TBP)**	Reverse	TATA-box binding protein	5’-ACCTTATAGGAAACTTCACATCAC-3’

Figure S1

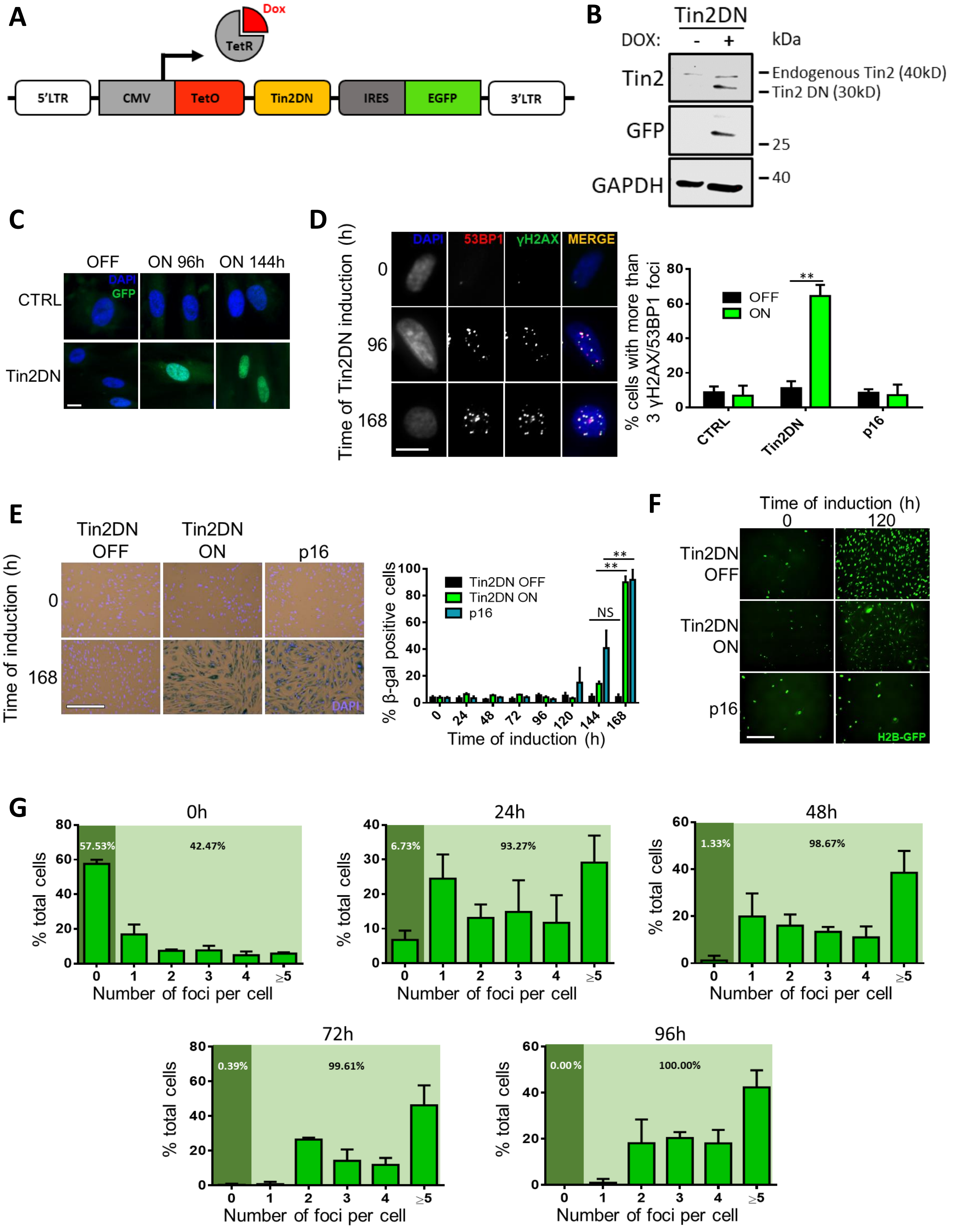

A
Dox
TetR
5'LTR
CMV
TetO
Tin2DN
IRES
EGFP
3'LTR
B
Tin2DN
DOX: - +
kDa
Tin2
GFP
GAPDH
Endogenous Tin2 (40kD)
Tin2 DN (30kD)
25
40
C
OFF
ON 96h
ON 144h
CTRL
Tin2DN
DAPI
GFP
D
Time of Tin2DN induction (h)
0
96
168
DAPI
53BP1
γH2AX
MERGE
% cells with more than 3 γH2AX/53BP1 foci
OFF
ON
CTRL
Tin2DN
p16
**
E
Tin2DN OFF
Tin2DN ON
p16
Time of induction (h)
0
168
DAPI
% β-gal positive cells
Time of induction (h)
NS
**
F
Time of induction (h)
0
120
Tin2DN OFF
Tin2DN ON
p16
H2B-GFP
G
0h
24h
48h
72h
96h
% total cells
Number of foci per cell
57.53%
42.47%
6.73%
93.27%
1.33%
98.67%
0.39%
99.61%
0.00%
100.00%

Figure S2

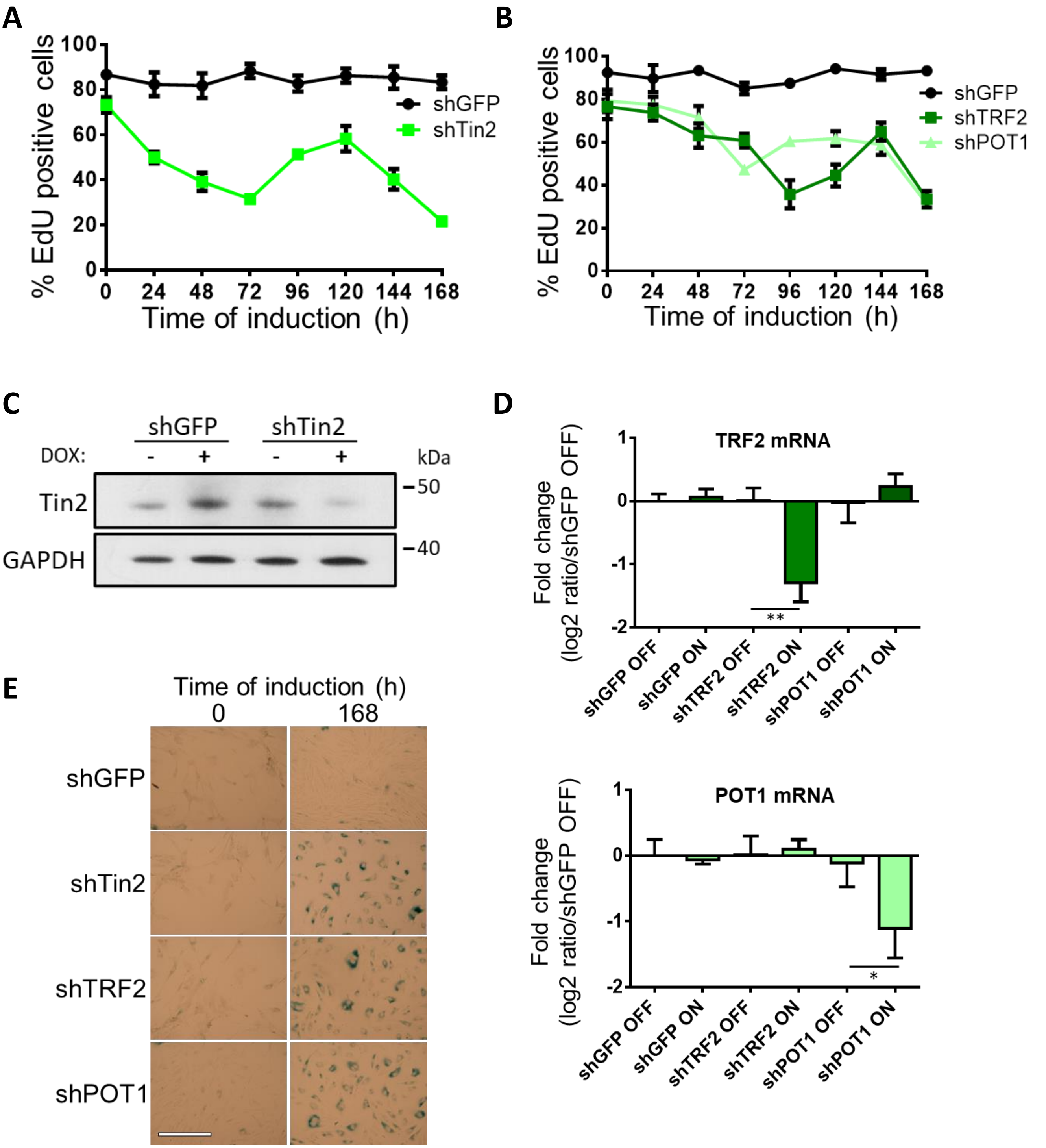
A
% EdU positive cells
100
80
60
40
20
0
0 24 48 72 96 120 144 168
Time of induction (h)
shGFP
shTin2
B
% EdU positive cells
Time of induction (h)
shGFP
shTRF2
shPOT1
C
shGFP
shTin2
DOX: - + - +
kDa
Tin2
GAPDH
50
40
D
TRF2 mRNA
Fold change (log2 ratio/shGFP OFF)
**
shGFP OFF
shGFP ON
shTRF2 OFF
shTRF2 ON
shPOT1 OFF
shPOT1 ON
POT1 mRNA
*
E
Time of induction (h)
0
168
shGFP
shTin2
shTRF2
shPOT1

Figure S3

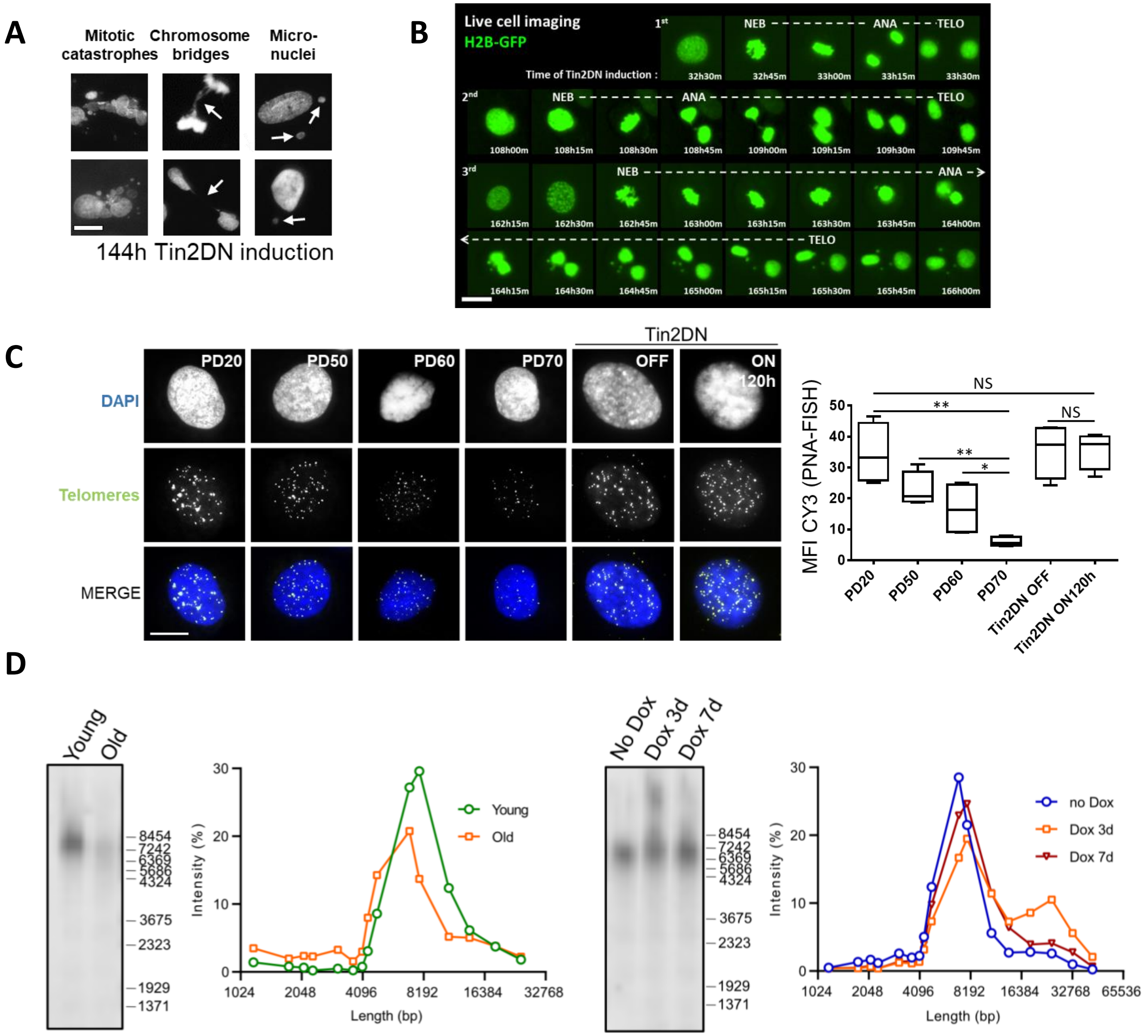
A
Mitotic catastrophes
Chromosome bridges
Micro-nuclei
144h Tin2DN induction
B
Live cell imaging
H2B-GFP
Time of Tin2DN induction :
1st
NEB
ANA
TELO
32h30m
32h45m
33h00m
33h15m
33h30m
2nd
108h00m
108h15m
108h30m
108h45m
109h00m
109h15m
109h30m
109h45m
3rd
162h15m
162h30m
162h45m
163h00m
163h15m
163h30m
163h45m
164h00m
164h15m
164h30m
164h45m
165h00m
165h15m
165h30m
165h45m
166h00m
C
Tin2DN
PD20
PD50
PD60
PD70
OFF
ON 120h
DAPI
Telomeres
MERGE
MFI CY3 (PNA-FISH)
NS
**
**
*
Tin2DN OFF
Tin2DN ON120h
D
Young
Old
8454
7242
6369
5686
4324
3675
2323
1929
1371
Intensity (%)
Length (bp)
No Dox
Dox 3d
Dox 7d
no Dox
1024
2048
4096
8192
16384
32768
65536

Figure S3 continued

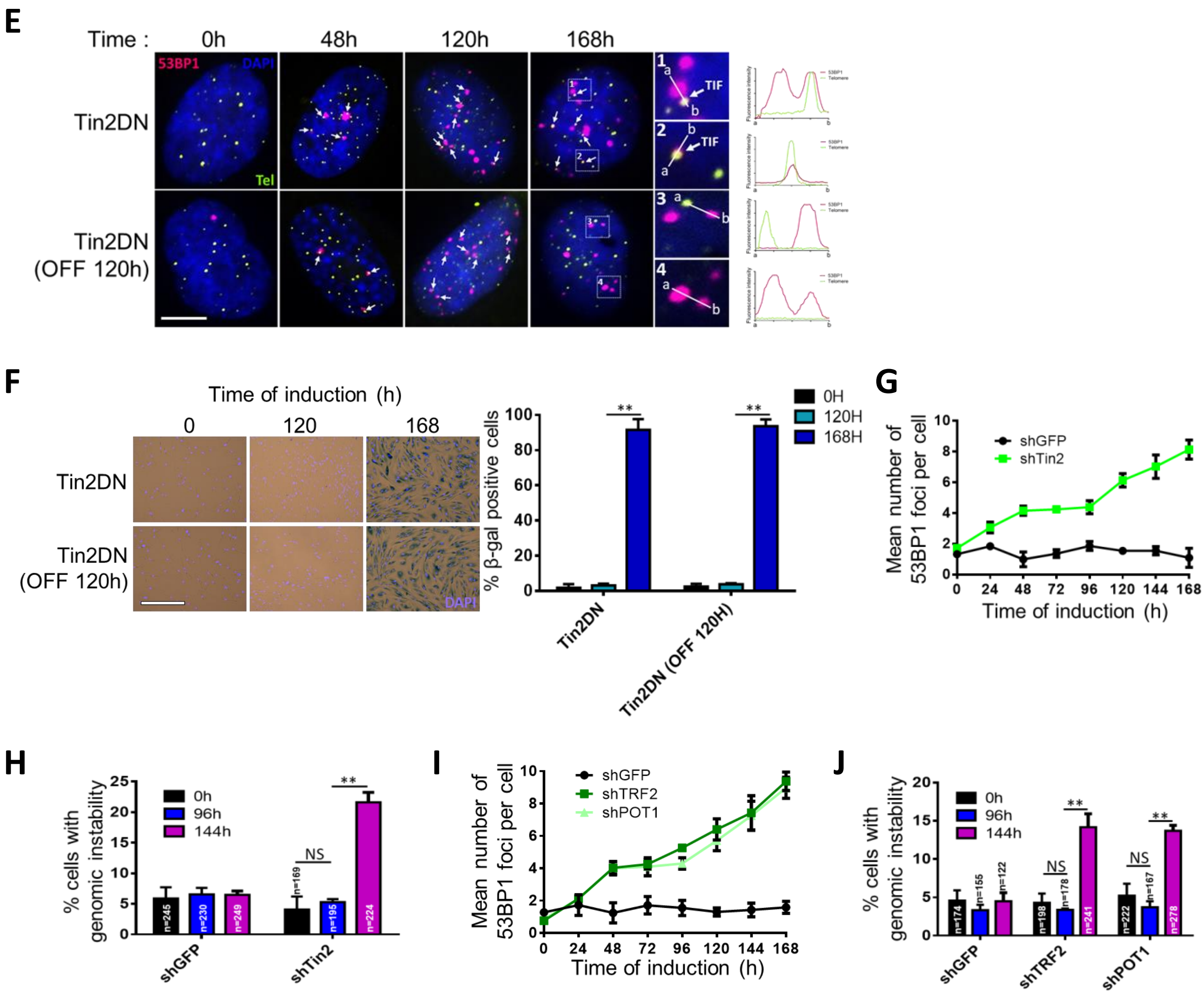

Figure S4

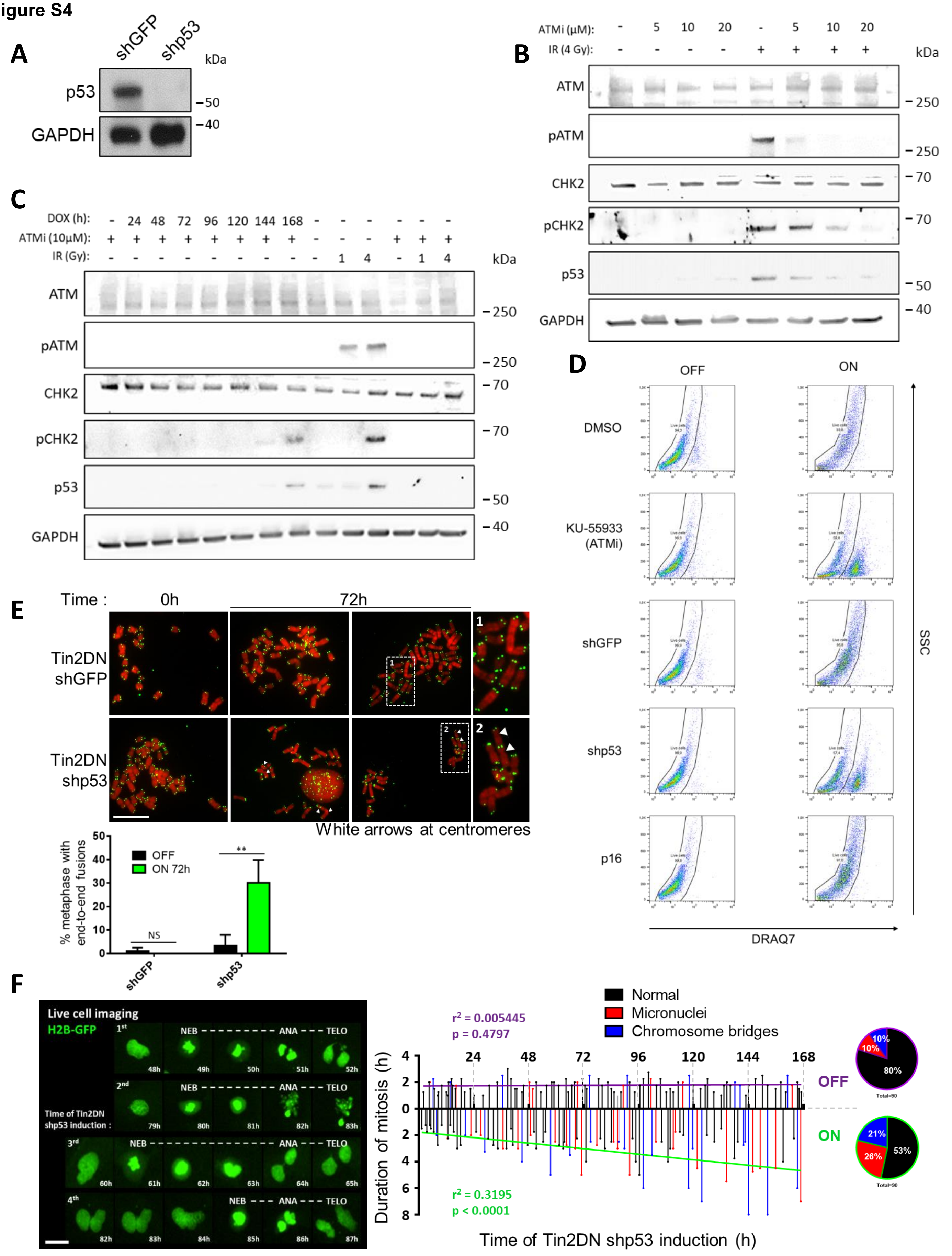
A
shGFP
shp53
kDa
p53
GAPDH
50
40
B
ATMi (µM): - 5 10 20 - 5 10 20
IR (4 Gy): - - - - + + + +
kDa
ATM
pATM
CHK2
pCHK2
p53
GAPDH
250
250
70
70
50
40
C
DOX (h): - 24 48 72 96 120 144 168 - - - - - -
ATMi (10µM): + + + + + + + + - - - + + +
IR (Gy): - - - - - - - - - 1 4 - 1 4
kDa
ATM
pATM
CHK2
pCHK2
p53
GAPDH
250
250
70
70
50
40
D
OFF
ON
DMSO
KU-55933 (ATMi)
shGFP
shp53
p16
SSC
DRAQ7
E
Time :
0h
72h
Tin2DN shGFP
Tin2DN shp53
White arrows at centromeres
% metaphase with end-to-end fusions
OFF
ON 72h
NS
**
shGFP
shp53
F
Live cell imaging
H2B-GFP
NEB
ANA
TELO
Time of Tin2DN shp53 induction :
Normal
Micronuclei
Chromosome bridges
r² = 0.005445
p = 0.4797
r² = 0.3195
p < 0.0001
OFF
ON
Duration of mitosis (h)
Time of Tin2DN shp53 induction (h)
80%
10%
10%
53%
26%
21%
Total=90
Total=90

Figure S5

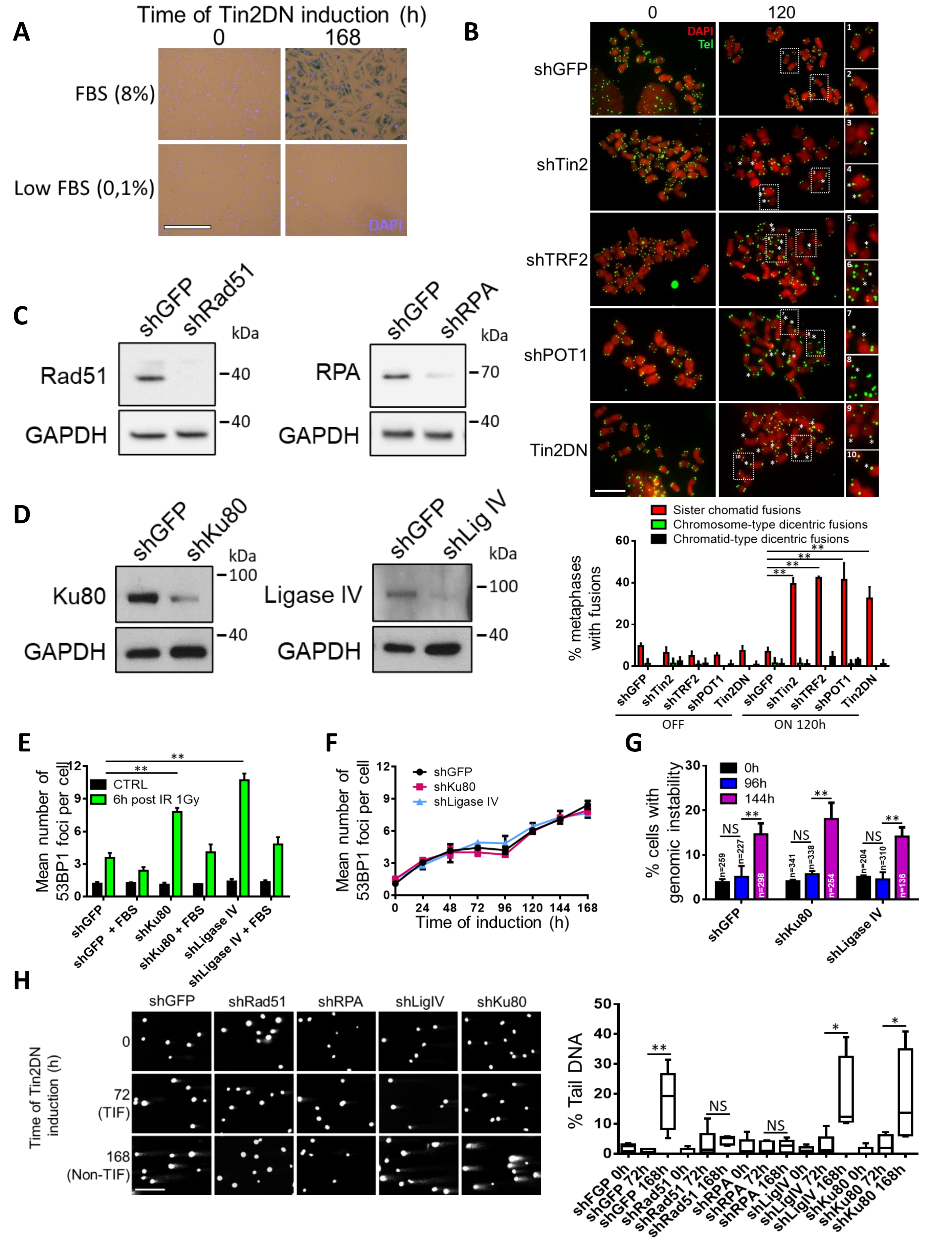
A
Time of Tin2DN induction (h)
0
168
FBS (8%)
Low FBS (0,1%)
DAPI
B
Time of induction (h)
0
120
DAPI
Tel
shGFP
shTin2
shTRF2
shPOT1
Tin2DN
Sister chomatid fusions
Chromosome-type dicentric fusions
Chromatid-type dicentric fusions
% metaphases with fusions
OFF
ON 120h
C
shGFP
shRad51
kDa
Rad51
GAPDH
40
shRPA
RPA
70
D
shKu80
Ku80
100
shLig IV
Ligase IV
E
Mean number of 53BP1 foci per cell
CTRL
6h post IR 1Gy
shGFP
shGFP + FBS
shKu80
shKu80 + FBS
shLigase IV
shLigase IV + FBS
F
shGFP
shKu80
shLigase IV
Time of induction (h)
G
% cells with genomic instability
0h
96h
144h
NS
H
shGFP
shRad51
shRPA
shLigIV
shKu80
Time of Tin2DN induction (h)
72 (TIF)
168 (Non-TIF)
% Tail DNA
shFGP 0h
shGFP 72h
shGFP 168h
shRad51 0h
shRad51 72h
shRad51 168h
shRPA 0h
shRPA 72h
shRPA 168h
shLigIV 0h
shLigIV 72h
shLigIV 168h
shKu80 0h
shKu80 72h
shKu80 168h

Figure S6

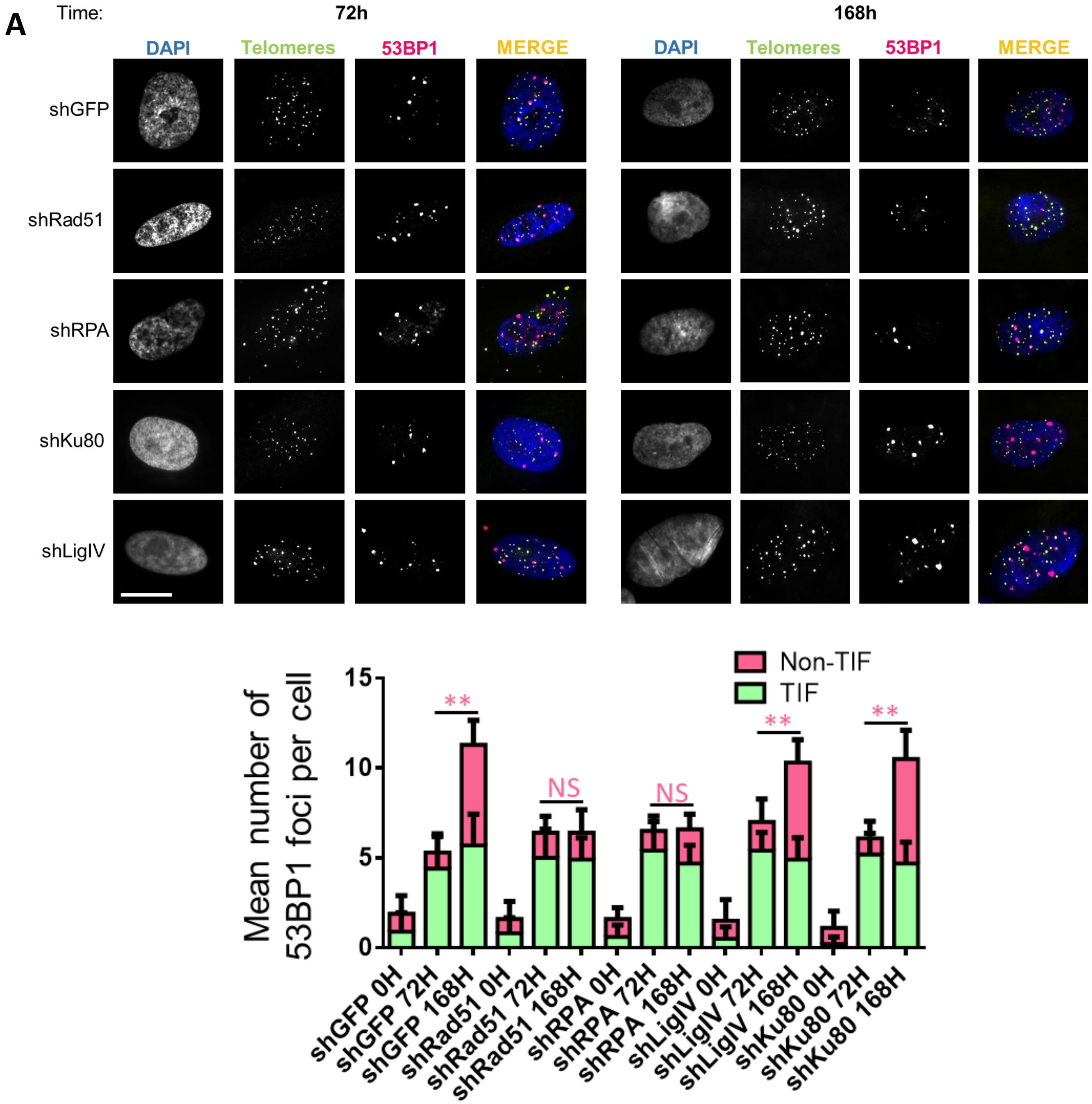
A
Time:
72h
168h
DAPI
Telomeres
53BP1
MERGE
shGFP
shRad51
shRPA
shKu80
shLigIV
Non-TIF
TIF
Mean number of 53BP1 foci per cell
15
10
5
0
**
NS
shGFP 0H
shGFP 72H
shGFP 168H
shRad51 0H
shRad51 72H
shRad51 168H
shRPA 0H
shRPA 72H
shRPA 168H
shLigIV 0H
shLigIV 72H
shLigIV 168H
shKu80 0H
shKu80 72H
shKu80 168H

Figure S7

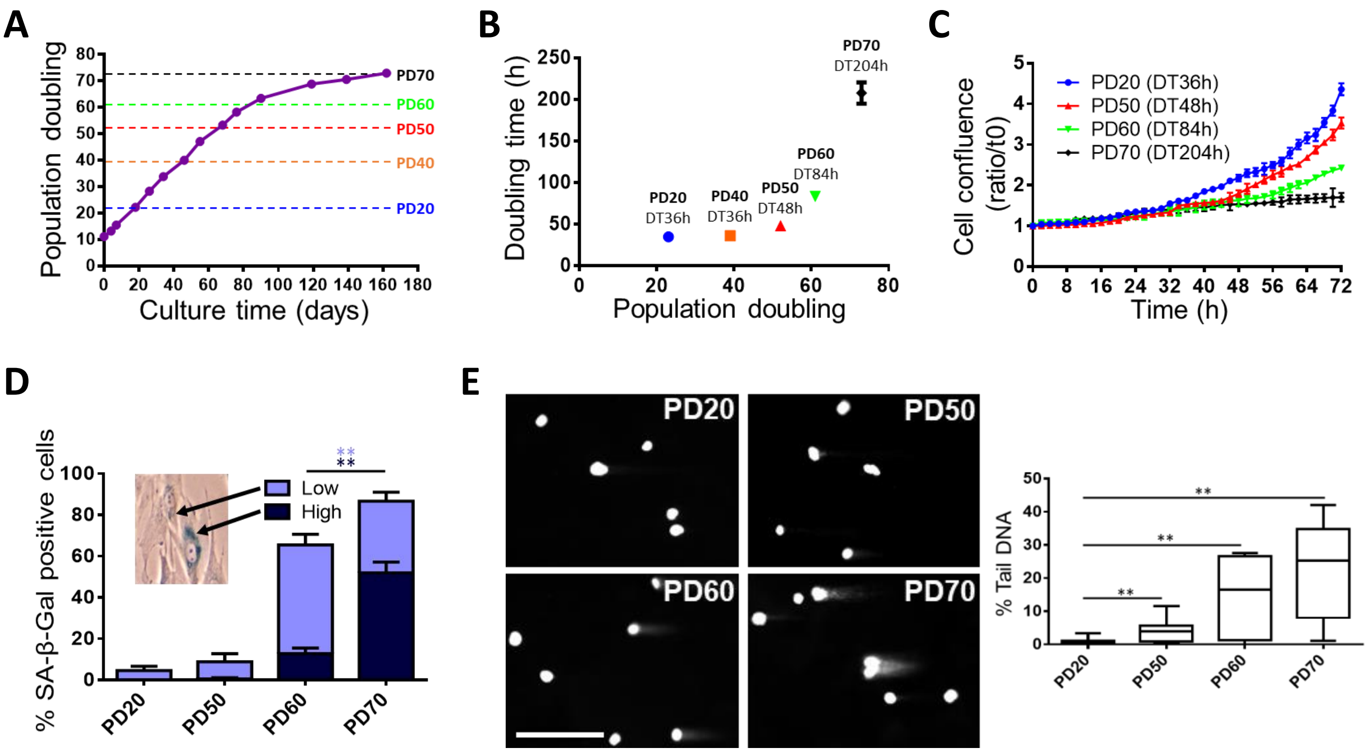
A
Population doubling
Culture time (days)
PD70
PD60
PD50
PD40
PD20
B
Doubling time (h)
Population doubling
PD70
DT204h
PD60
DT84h
PD50
DT48h
PD40
DT36h
PD20
DT36h
C
Cell confluence (ratio/t0)
Time (h)
PD20 (DT36h)
PD50 (DT48h)
PD60 (DT84h)
PD70 (DT204h)
D
% SA-β-Gal positive cells
Low
High
PD20
PD50
PD60
PD70
E
PD20
PD50
PD60
PD70
% Tail DNA
PD20
PD50
PD60
PD70

Figure S8

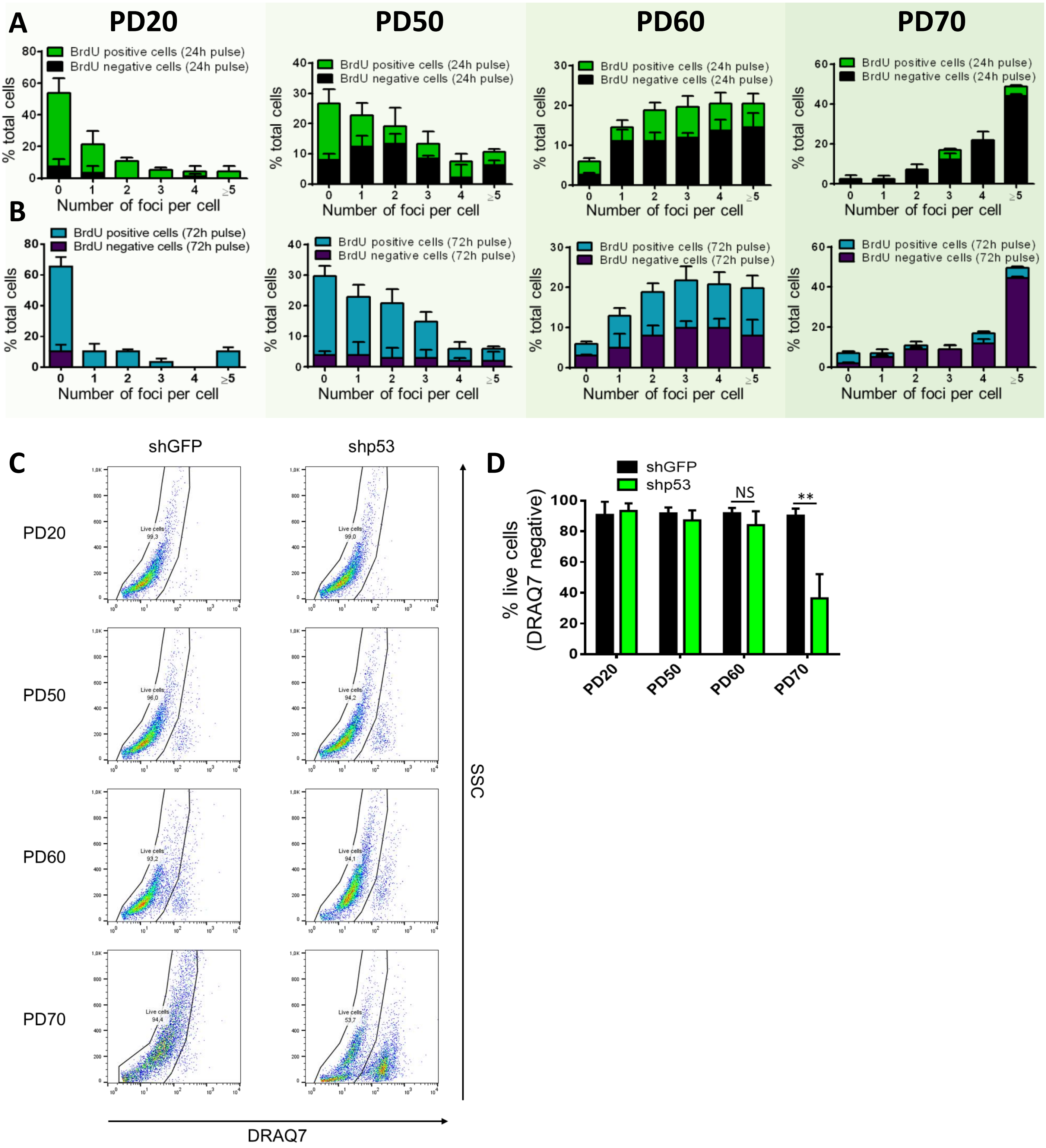
A
B
PD20
PD50
PD60
PD70
BrdU positive cells (24h pulse)
BrdU negative cells (24h pulse)
BrdU positive cells (72h pulse)
BrdU negative cells (72h pulse)
% total cells
Number of foci per cell
C
shGFP
shp53
Live cells
SSC
DRAQ7
D
% live cells (DRAQ7 negative)
NS
**
